# Integrating Competitive Li^+^ Coordination with Immobilized Anions in Composite Solid Electrolyte for High‐Performance Li Metal Batteries

**DOI:** 10.1002/advs.202413875

**Published:** 2025-02-18

**Authors:** Ziyang Liang, Chang Liu, Xiang Bai, Jiahui Zhang, Xinyue Chang, Bo Zhang, Mengxue Xia, Huayun Du, Hao Huang, Bing Wu, Chengkai Yang, Shi Wang, Wen Liu, Qian Wang

**Affiliations:** ^1^ College of Materials Science and Engineering Taiyuan University of Technology Taiyuan Shanxi 030024 China; ^2^ Shanxi Energy Internet Research Institute Taiyuan Shanxi 030024 China; ^3^ Emergency Research Institute Chinese Institute of Coal Science (CICS) Beijing 100013 China; ^4^ Key Laboratory of Advanced Materials Technology College of Materials Science and Engineering Fuzhou University Fuzhou 350108 China; ^5^ State Key Laboratory of Organic Electronics & Information Displays (SKLOEID) Institute of Advanced Materials (IAM) Nanjing University of Posts & Telecommunications Nanjing 210023 China; ^6^ State Key Lab of Chemical Resource Engineering College of Science and College of Energy Beijing University of Chemical Technology Beijing 100092 China

**Keywords:** composite solid electrolytes, immobilized anions, Li metal batteries, Li^+^ coordination, ZIF‐90‐NH_2_

## Abstract

Poly(vinylidene fluoride) (PVDF)‐based polymer electrolytes have attracted widespread attention due to their unique Li^+^ transport mechanism. However, their low ionic conductivity and porous structure, as well as residual solvent limit their application at high current densities. Here, a composite solid electrolyte (CSE) is developed by integrating poly(vinylidene‐co‐trifluoroethylene) [P(VDF‐TrFE)] in its all‐trans conformation with aminofunctionalized metal–organic framework (ZIF‐90‐NH_2_). In such a CSE, all F atoms located on one side of the polymer chain, providing fast Li^+^ transport channels. Concurrently, the functionalized ZIF‐90‐NH_2_ can effectively anchor the residual N, N‐dimethylformamide (DMF) in CSEs while weakening Li^+^‐DMF solvent coordination, inducing the rearrangement of Li^+^ solvation structure and inhibiting the decomposition of DMF at the interface. Synergistically, ZIF‐90‐NH_2_ can immobilize anions in Li salts, promoting their dissociation. Based on integrating competitive Li^+^ coordination with immobilized anions, the obtained CSEs exhibit a high Li^+^ transference number (0.77). The full cells with LiFePO_4_ cathode can run stably over 400 cycles at 5 C, while the Li || LiNi_0.7_Co_0.1_Mn_0.2_O_2_ full cells deliver a high capacity retention (>85%) after 200 cycles at a charge cutoff voltage of 4.5 V. This work opens up a new path for building CSEs with high interfacial stability and fast Li^+^ transport.

## Graphical abstract

Amino‐functionalized metal–organic framework (ZIF‐90‐NH_2_) can adsorb DMF solvent molecules and Li salt anions, weakening the ion‐solvent coordination and inducing rearrangement of Li^+^ solvated structures, while facilitating the dissociation of Li salts. The composite solid electrolyte after combining ZIF‐90‐NH_2_ with poly(vinylidene‐co‐trifluoroethylene) in all‐trans conformation exhibits high ionic conductivity (6.05 × 10^−4^ S cm^−1^) and Li^+^ transference number (0.77).

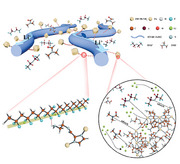

## 1 Introduction

Lithium‐ion batteries (LIBs) have been widely used in consumer electronics and electric vehicles.^[^ ^1^ ^]^ However, the current battery systems struggle to satisfy consumers' ongoing demand for high energy density and safety.^[^ ^2^ ^]^ Among various new battery systems, Li metal batteries (LMBs) have attracted widespread attention due to their high energy density.^[^ ^3^ ^]^ Especially the LMBs based on solid‐state electrolytes are regarded as one of the most promising next‐generation battery systems.^[^ ^4^ ^]^

Among the solid‐state electrolytes, solid polymer electrolytes (SPEs) deliver great application potential due to their high flexibility and interfacial compatibility.^[^ ^5^ ^]^ Up to now, a large number of SPEs systems have been developed, such as poly(ethylene oxide) (PEO)‐based SPEs,^[^ ^6^ ^]^ polyethylene‐based SPEs,^[^ ^7^ ^]^ polyacrylonitrile‐based SPEs,^[^ ^8^ ^]^ poly(vinylidene fluoride) (PVDF)‐based SPEs,^[^ ^9^ ^]^ poly(propylene carbonate)‐based SPEs,^[^ ^10^ ^]^ etc. Among them, the PVDF‐based SPEs have superior thermal stability, mechanical properties, wide electrochemical window, and unique Li^+^ transport mechanism, making them compatible with high‐voltage cathode materials, thus becoming particularly attractive in recent years.^[^ ^11^ ^]^ The residual N, N‐dimethylformamide (DMF) solvent in PVDF‐based SPEs can form [Li(DMF)_x_]^+^ complex structures, enabling Li^+^ transport through their interactions with F atoms on polymer chains.^[^ ^12^ ^]^ What gains from this is destroyed by this, which also brings several issues: i) The residual DMF solvent will also cause irreversible side reactions with Li metal anode, which gradually thicken the solid electrolyte interface (SEI) during cycling, resulting in increased interfacial impedance and dendritic Li growth.^[^ ^13^ ^]^ ii) The poor antioxidation capacity of DMF reduces the electrochemical window of SPEs.^[^ ^14^ ^]^ iii) The porous structure of PVDF‐based polymers leads to uneven Li^+^‐flux distribution across the SPEs, inducing uneven Li deposition and dendritic Li formation.^[^ ^15^ ^]^ iv) PVDF has a weak ability to dissociate Li salts and still suffers from low ionic conductivity.^[^ ^16^ ^]^

Tremendous efforts have been devoted to addressing these issues and the most typical strategy is to construct composite solid electrolytes (CSEs) by doping inorganic ceramic fillers,^[^ ^17^ ^]^ such as Li_7_La_3_Zr_2_O_12_,^[^ ^18^ ^]^ layered graphene,^[^ ^19^ ^]^ SiO_2_,^[^ ^20^ ^]^ Li_1.4_Al_0.4_Ti_1.6_(PO_4_)_3_,^[^ ^21^ ^]^ etc. Although introducing these inorganic fillers has improved the physicochemical properties of SPEs, such as ionic conductivity and mechanical strength, they have not effectively anchored residual DMF molecules, meaning that this does not fundamentally alter the solvation structure of [Li(DMF)_x_]^+^.^[^ ^22^ ^]^ In other words, the side reactions still cannot be effectively inhibited, which leads to poor interface stability, especially at high current density.^[^ ^23^ ^]^ Recently, Zhou et al. proposed a solvation tailoring strategy for the first time by introducing 3 Å zeolite molecular sieves into PVDF electrolytes to confine residual DMF molecules.^[^ ^24^ ^]^ The strong interaction between DMF and the molecular sieves weakens DMF's ability to participate in Li^+^‐solvation, leading to more anions engaging in solvation and consequently achieving a highly stable SEI layer. Nevertheless, the conformational arrangement of molecular chains in PVDF is predominantly cis‐trans alternating, which is detrimental to the rapid Li^+^ migration.^[^ ^25^ ^]^ Research has shown that poly(vinylidene‐co‐trifluoroethylene) [P(VDF‐TrFE)] with all‐trans conformation can provide rapid Li^+^ channels, but it exhibits a relatively low dielectric constant, which restricts Li salt dissociation, making it challenging to achieve exceptionally high ionic conductivity in the resulting SPEs. Thus, developing innovative strategies to further regulate the solvation structure of P(VDF‐TrFE)–based SPEs and promote the dissociation of Li salts is vital for the practical application of fluorinated polymer electrolytes. This will also provide new solutions to the current bottlenecks faced by polymer electrolytes. Metal–organic frameworks (MOFs) with high specific surface area and strong adsorption have attracted our attention.^[^ ^26^ ^]^

In this work, a functionalized metal–organic framework (ZIF‐90‐NH_2_) was introduced to P(VDF‐TrFE) electrolyte to solve the above challenges. The aminofunctionalized ZIF‐90‐NH_2_ displayed a strong interaction with DMF molecules in SPEs by ion‐dipole interactions, weakening the binding strength of Li^+^‐O in the solvation structure of [Li(DMF)_x_]^+^ and regulating the Li^+^ competitive coordination, thus inhibiting the decomposition of DMF at the interface and further promoting the Li^+^ migration. Synergistically, it also strongly immobilized the anions in Li salts by electrostatic interactions, promoting their dissociation and increasing the Li^+^ transference number. Lastly, a LiF‐rich SEI layer can be formed on the surface of Li metal anode. Thus, the Li || Li symmetric cell can be stably cycled for 1000 h at 0.2 mA cm^−2^ and maintain a polarization voltage of 30 mV. Li || LiFePO_4_ full cells can run stably over 400 cycles at a higher rate of 5 C. The Li || LiNi_0.7_Co_0.1_Mn_0.2_O_2_ full cells deliver a high capacity retention (>85%) after 200 cycles at a high charge cutoff voltage of 4.5 V.

## 2 Results and Discussion

### 2.1 Design Concept and Preparation of CSEs

**Figure 1:**
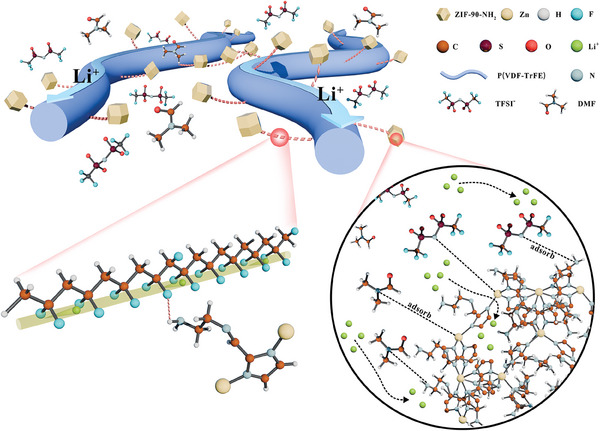
Schematic diagram of the ordered arrangement structure of PTZN electrolyte and immobilization of DMF and TFSI^−^ anions by functionalized ZIF‐90‐NH_2_.

To construct CSEs, we first prepared aminated ZIF‐90‐NH_2_. As shown in **Figure** **1**, ZIF‐90‐NH_2_ with Lewis acidic Zn metal sites and positively charged ‐NH_2_ groups can effectively adsorb DMF solvent molecules via ion‐dipole interactions, while the anion of Li salt (TFSI^−^) is also firmly anchored by electrostatic interactions, thus significantly promoting the dissociation of Li salts and regulating the solvation structure of [Li(DMF)_x_]^+^. Meanwhile, its dodecahedral and porous structure also an extensive interaction surface with Li salts and DMF molecules, facilitating rapid Li^+^ transport along the ZIF‐90‐NH_2_ interface and within the pores. Moreover, P(VDF‐TrFE) has an all‐trans conformation where all F atoms are uniformly arranged on the same side of the C chain, thus forming a highway for Li^+^ transport.^[^ ^27^ ^]^ Additionally, the F atoms in P(VDF‐TrFE) can form hydrogen bonding interactions with ‐NH_2_ in ZIF‐90‐NH_2_, which improves the antioxidant properties of CSEs and reduces the polymer chain entanglement, thus constructing a stable Li^+^ transport channel.

**Figure 2:**
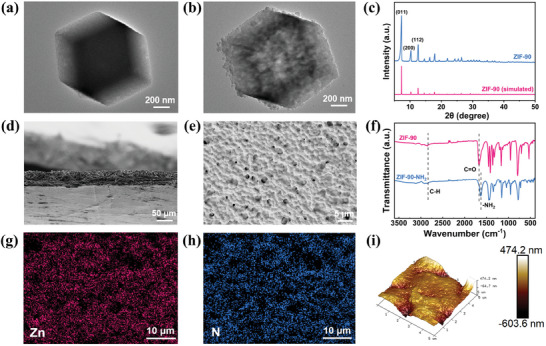
Preparation and analysis of CSEs. Transmission electron microscopy images of a) ZIF‐90 and b) ZIF‐90‐NH_2_. c) XRD spectra of ZIF‐90 and simulated ZIF‐90. d) Cross section and e) surface scanning electron microscopy (SEM) images of PTZN electrolyte. f) FTIR spectra of ZIF‐90 and ZIF‐90‐NH_2_. g,h) EDS mapping of PTZN electrolyte. i) Atomic force microscopy (AFM) images of PTZN electrolyte.

Specifically, we first synthesized ZIF‐90 with a standard dodecahedral structure and a particle size of ≈1.0 µm. After ‐NH_2_ modification, its surface showed a certain degree of roughness and there was no change in structure and size (**Figure** **2**a,b; Figure S1, Supporting Information). The X‐ray diffraction (XRD) pattern showed that the diffraction peaks of the synthesized ZIF‐90 at 2θ = 7.2°, 10.2°, and 12.6° correspond to the (011), (200), and (112) crystal planes, respectively, proving the successful synthesis of ZIF‐90 (Figure 2c). Meanwhile, the Fourier transform infrared (FTIR) spectrum displayed that the characteristic peak at 2830 cm^−1^ corresponds to the C─H bond of the acetaldehyde group, while the peak at 1672 cm^−1^ corresponds to the C ═ O stretching vibration within the acetaldehyde group.^[^ ^28^ ^]^ The characteristic peak at 1635 cm^−1^ can be attributed to the stretching vibration of the ‐NH_2_ group (Figure 2f; Figure S2, Supporting Information). Compared to ZIF‐90, the disappearance of the C ═ O peak and the appearance of the ‐NH_2_ peak in ZIF‐90‐NH_2_ demonstrated that the aldehyde group in ZIF‐90 was successfully replaced by ethylenediamine, confirming the successful synthesis of ZIF‐90‐NH_2_. Furthermore, ZIF‐90‐NH_2_ exhibited a remarkably high specific surface area of 1262.46 m^2^ g^−1^ and an average pore size of 4.02 nm (Figures S3 and S4, Supporting Information). The larger specific surface area and pore size facilitate the absorption and immobilization of more TFSI^−^ anions and DMF solvent molecules, thereby enhancing the Li⁺ transference number and stabilizing the anode/electrolyte interface.

Then, we combined ZIF‐90‐NH_2_ with P(VDF‐TrFE) to prepare CSEs (named: PTZN electrolyte). As shown in Figure 2d,e and Figure S5 (Supporting Information), in comparison to the bare P(VDF‐TrFE) electrolyte, the PTZN electrolyte had a smoother, flatter surface and a dense, compact framework, which was beneficial for improving the interface contact and facilitating Li^+^ transport. Energy dispersive spectroscopy (EDS) mapping showed that the ZIF‐90‐NH_2_ particles were uniformly dispersed in the PTZN electrolyte, which was attributed to the strong hydrogen bonding between P(VDF‐TrFE) and ZIF‐90‐NH_2_ (Figure 2g,h). Besides, the average roughness of the bare P(VDF‐TrFE) electrolyte surface was notably high at 532 nm, while after doping with ZIF‐90‐NH_2_, the average roughness of PTZN electrolyte was significantly reduced to 84 nm. Additionally, the thickness of the PTZN electrolyte was only 60 µm, compared to 65 µm for the P(VDF‐TrFE) electrolyte, which effectively shortened the Li^+^ transport distance (Figure 2i; Figures S6 and S7a, Supporting Information). Meanwhile, the Young's modulus of the PTZN electrolyte was 580 MPa, surpassing that of the bare P(VDF‐TrFE) electrolyte (Figure S7b,c, Supporting Information).

**Figure 3:**
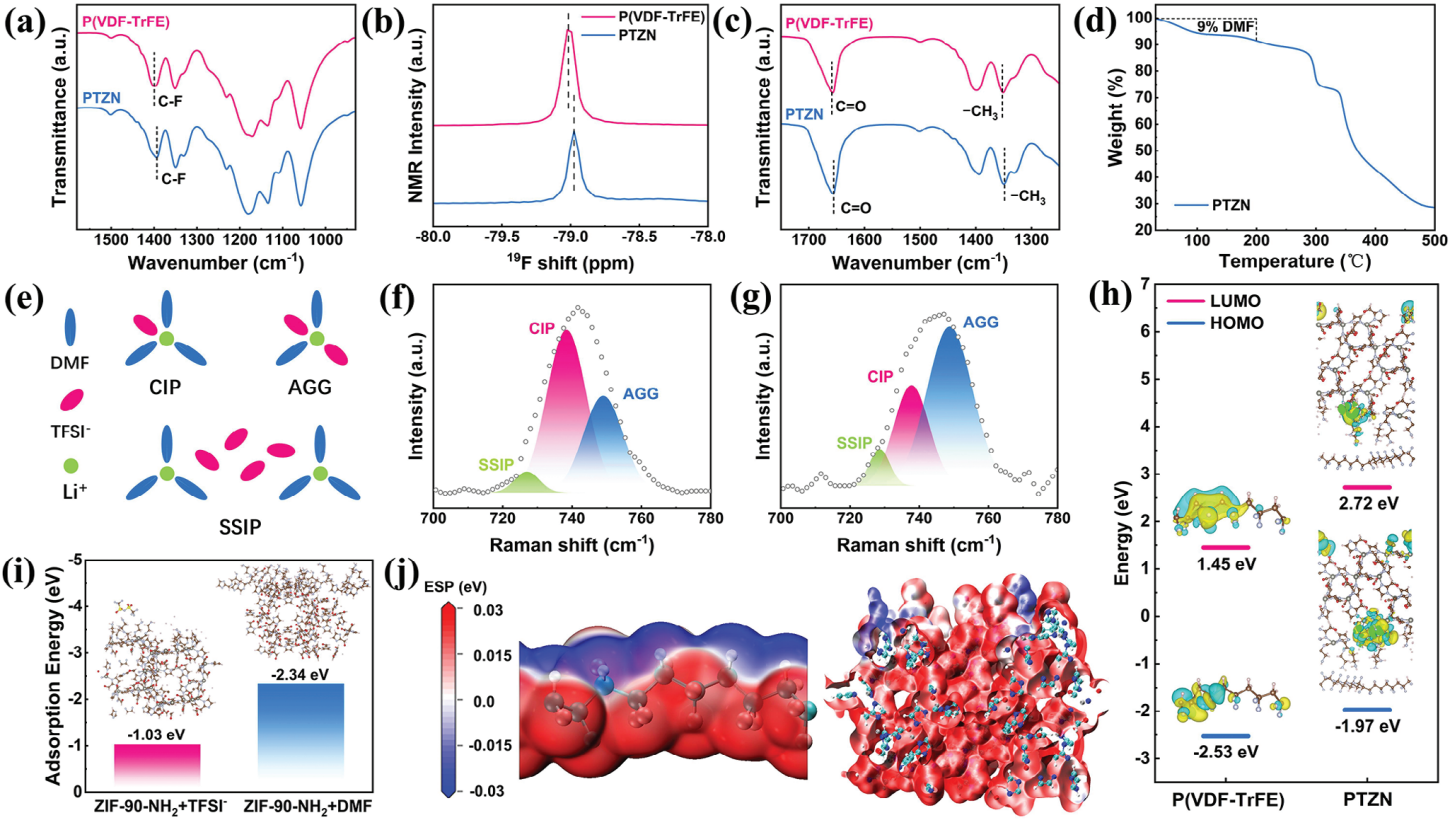
Characterization and DFT calculations of the CSEs. a) FTIR spectra and b) solid‐state ^19^F NMR spectra of P(VDF‐TrFE) and PTZN electrolytes. c) FTIR spectra of P(VDF‐TrFE) and PTZN electrolytes in different ranges. d) TGA curves of PTZN electrolytes before cycling. e) Schematic diagram of solvation structure. Raman spectra of f) P(VDF‐TrFE) and g) PTZN electrolytes. h) LUMO and HOMO energy levels of the electrolytes. i) Adsorption energy calculation of TFSI anions and DMF molecules on ZIF‐90‐NH_2_. j) The electrostatic potentials of P(VDF‐TrFE) and ZIF‐90‐NH_2_.

Subsequently, we analyzed the interaction mechanisms between P(VDF‐TrFE), LiTFSI, DMF and ZIF‐90‐NH_2_ using FTIR, nuclear magnetic resonance (NMR), and Raman. As shown in **Figure** **3**a, the peak at 1398 cm^−1^ in P(VDF‐TrFE) electrolyte corresponded to the bending vibration of C─F bonds. After introducing ZIF‐90‐NH_2_, this C‐F peak experienced a red shift from 1398 to 1393 cm^−1^, indicating the formation of hydrogen bonding interactions between P(VDF‐TrFE) and ZIF‐90‐NH_2_. Meanwhile, the ^19^F NMR profiles of P(VDF‐TrFE) and PTZN electrolytes also demonstrated that the peaks corresponding to C─F bonds shift to a lower frequency upon the addition of ZIF‐90‐NH_2_, further suggesting the formation of hydrogen bonding (Figure 3b). Moreover, the peaks at 1658 and 1351 cm^−1^, corresponding to C ═ O and ‐CH_3_ in the DMF solvent, respectively, exhibited a red shift to 1655 and 1349 cm^−1^ after the introduction of ZIF‐90‐NH_2_ particles, which indicated the strong interaction between DMF and ZIF‐90‐NH_2_ (Figure 3c). Notably, the DMF eigenpeak signals in PTZN were stronger than those in the P(VDF‐TrFE) electrolyte, indicating that the coordination of Li^+^ to DMF was weaker in the PTZN electrolyte (Figure S8, Supporting Information).^[^ ^24^ ^]^ Furthermore, thermogravimetric analysis (TGA) results for the PTZN electrolyte revealed that the residual DMF solvent content was 8.9 wt% before cycling and remained almost unchanged after 50 cycles (Figure 3d; Figure S9, Supporting Information). This phenomenon underscored that most of the DMF solvent was immobilized by the Zn metal sites and positively charged ‐NH_2_ groups in ZIF‐90‐NH_2_, thereby reducing the side reactions between DMF and Li metal anode during cycling. Raman spectroscopy also proved these findings, which is shown in Figure 3e–g. The peaks at 725, 737, and 748 cm^−1^ correspond to SSIP (free TFSI^−^ anion), CIP (one TFSI^−^ coordinated to Li^+^), and AGG (multiple TFSI^−^ coordinated to Li^+^), respectively.^[^ ^29^ ^]^ Specifically, the proportion of SSIP increased from 4.8% to 6.6% after introducing ZIF‐90‐NH_2_, which demonstrated that the electrostatic interaction between ZIF‐90‐NH_2_ and TFSI^−^ promoted the dissociation of Li salts. Moreover, the proportion of CIP decreased from 59.7% to 30.7%, and the content of AGG increased dramatically from 35.5% to 62.7%, which suggested that the strong adsorption of ZIF‐90‐NH_2_ weakened the DMF‐Li^+^ interaction and lowered the ability of DMF to participate in solvation, thus inducing a rearrangement of the Li^+^ solvated structure.

In addition, in order to further confirm the interaction between CSEs, LiTFSI, DMF, and ZIF‐90‐NH_2_, we also performed density functional theory (DFT) calculations. As shown in Figure 3j and Figure S10 (Supporting Information), the Zn metal sites and ‐NH_2_ group in ZIF‐90‐NH_2_ had a large number of positive charges, which could combine with the strongly electronegative F atoms in P(VDF‐TrFE) to form hydrogen bonds, and the calculated binding energy was 0.34 eV, which indicated that the structural stability of PTZN electrolyte was improved. Moreover, thanks to hydrogen bonding, the HOMO and LUMO energy levels increased with the addition of ZIF‐90‐NH_2_, which improved the antioxidant property of the electrolyte (Figure 3h). As shown in Figure 3i and Figure S11 (Supporting Information), the adsorption energy of DMF molecules on ZIF‐90‐NH_2_ was −2.34 eV, which was much larger than the adsorption energy of DMF molecules on P(VDF‐TrFE) (0.0036 eV), suggesting that the residual DMF molecules in PTZN electrolyte were more inclined to be adsorbed by ZIF‐90‐NH_2_, which weakened the interaction between Li^+^ and DMF in solvation structure and inhibited the occurrence of DMF side reactions at the interface. Meanwhile, ZIF‐90‐NH_2_ also had a strong adsorption effect on TFSI^−^, and its adsorption energy was calculated to be −1.03 eV, which further verified the anion immobilization effect of ZIF‐90‐NH_2_.

### 2.2 Physicochemical Properties of PTZN Electrolyte

**Figure 4:**
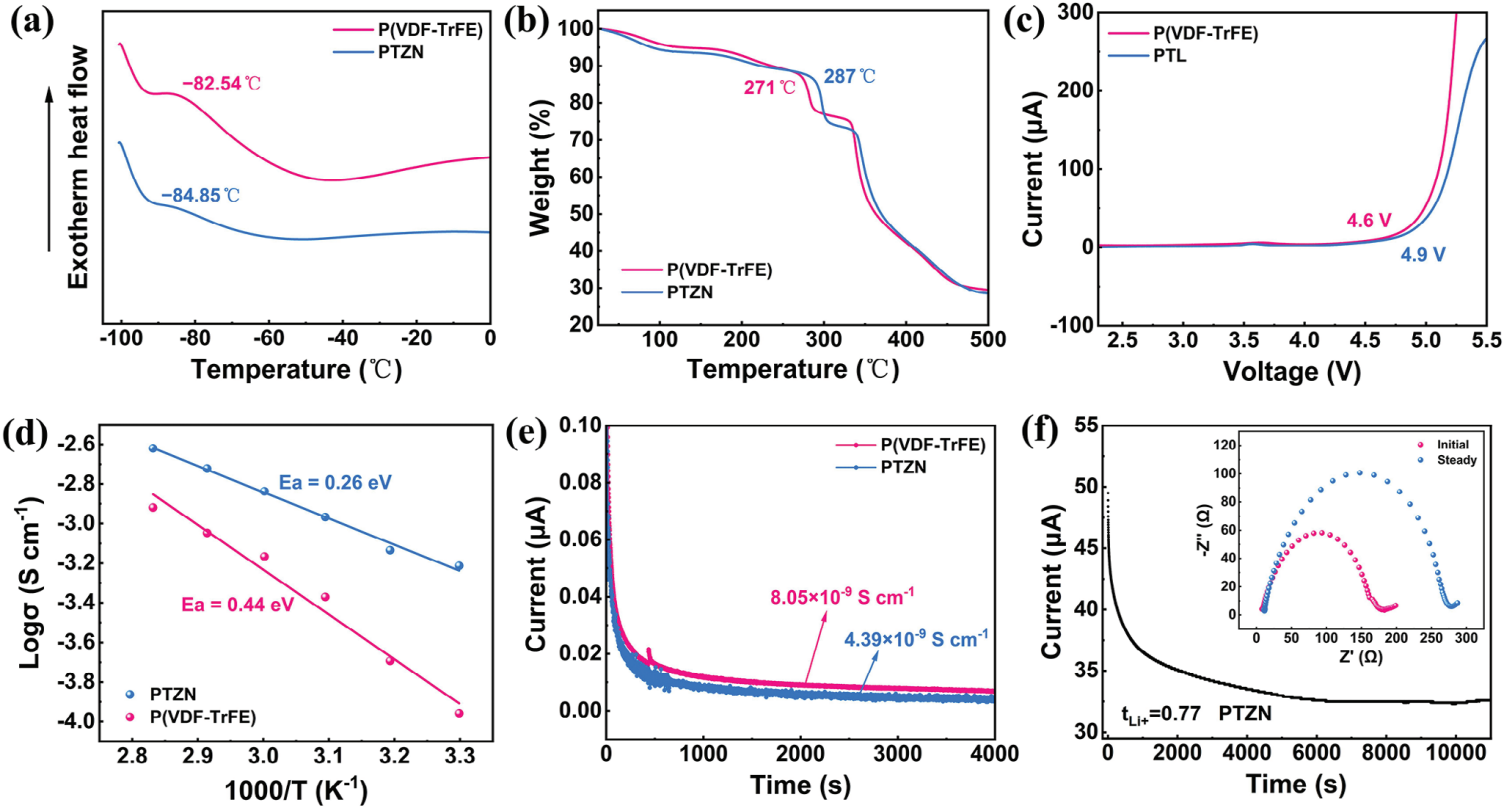
Physicochemical properties of CSEs. a) Differential scanning calorimetry and b) TGA curves of P(VDF‐TrFE) and PTZN electrolytes. c) Linear sweep voltammetry and d) Arrhenius curves of P(VDF‐TrFE) and PTZN electrolytes. e) Current‐time curves of P(VDF‐TrFE) and PTZN electrolytes at polarization voltage of 0.3 V. f) Current‐time curves of Li/PTZN/Li cells with insertion of initial versus steady state EIS spectra.

Generally speaking, lower the glass transition temperature (T_g_) facilitates Li^+^ migration. As shown in **Figure** **4**a, the T_g_ of the PTZN electrolyte was −84.85 °C, which was lower than that of the bare P(VDF‐TrFE) electrolyte (−82.54 °C), suggesting that the incorporation of ZIF‐90‐NH_2_ and the formation of hydrogen bonding reduced the polymer's crystallinity, thereby enhancing the mobility of polymer chain segments and facilitating Li^+^ transference. Additionally, the TGA results showed that the thermal decomposition temperature of CSEs increased from 271 °C to 287 °C after introducing ZIF‐90‐NH_2_, demonstrating enhanced thermal stability (Figure 4b). The thermal weight loss observed below 200 °C was attributed to the volatilization of DMF solvent. The residual DMF solvent content was almost the same for both electrolytes, which indicated that the high ionic conductivity of PTZN electrolyte was not related to the DMF solvent content.

After that, we investigated the electrochemical properties of the PTZN electrolyte. As shown in Figure 4c, the electrochemical window of PTZN electrolyte extended to 4.9 V, markedly surpassing the 4.6 V of P(VDF‐TrFE) electrolyte. This enhanced electrochemical stability can be attributed to the hydrogen‐bonding interactions between ZIF‐90‐NH_2_ and P(VDF‐TrFE). Then, we calculated the ionic conductivity according to the electrochemical impedance spectroscopy (EIS) at different temperatures. As shown in Figure S12 (Supporting Information), the ionic conductivity of PTZN electrolyte was as high as 6.05 × 10^−4^ S cm^−1^ at room temperature, which was 5.7 times higher than that of the P(VDF‐TrFE) electrolyte (1.06 × 10^−4^ S cm^−1^). This was attributed to the ordered arrangement of P(VDF‐TrFE) and ZIF‐90‐NH_2_ under hydrogen bonding, in synergy with the all‐trans conformation of the polymer, facilitating efficient and rapid Li^+^ transport. Besides, according to the Arrhenius equation, the activation energies (E_a_) of the P(VDF‐TrFE) and PTZN electrolytes were calculated to be 0.44 and 0.23 eV, respectively, suggesting that the incorporation of ZIF‐90‐NH_2_ reduced the migration energy barrier for Li^+^ (Figure 4d).^[^ ^30^ ^]^ The direct current polarisation test showed that the electronic conductivity of PTZN electrolyte was 4.39 × 10^−9^ S cm^−1^, which was lower than that of the P(VDF‐TrFE) electrolyte (8.05 × 10^−8^ S cm^−1^), indicating that PTZN had a good insulating property (Figure 4e).

The Li^+^ transference number is also a crucial parameter in solid‐state Li metal batteries. A higher Li^+^ transference number can inhibit the formation of space charge near Li metal anode and homogenize the electric field, thus facilitating uniform Li deposition.^[^ ^5b^ ^]^ As shown in Figure 4f, the Li^+^ transference number of PTZN electrolyte was 0.77, significantly exceeding that of the P(VDF‐TrFE) electrolyte. This can be attributed to the fact that under Lewis acid‐base and electrostatic interactions, ZIF‐90‐NH_2_ can adsorb the TFSI^−^ anions more easily, effectively promoting the dissociation of Li salts and releasing more free Li^+^. Our PTZN electrolyte had higher ionic conductivity and Li^+^ transference number compared to currently reported PVDF‐based composite solid electrolytes (Figure S13, Supporting Information).

### 2.3 Li Plating/Stripping Behavior and Post Analysis

**Figure 5:**
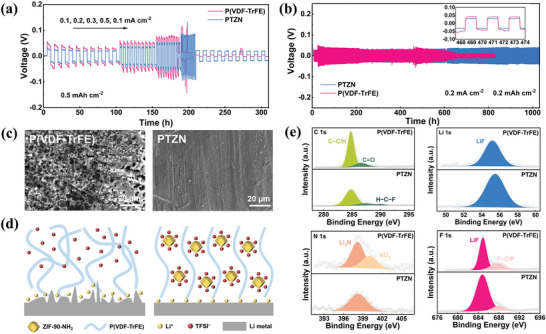
Symmetrical cells and post analysis. a) Rate performance of symmetrical cells with P(VDF‐TrFE) and PTZN electrolytes. b) Cycling test of symmetrical cells at a current density of 0.2 mA cm^−2^/0.2 mAh cm^−2^. c) SEM image of Li metal surface after cycling with P(VDF‐TrFE) and PTZN electrolytes. d) Schematic diagram of Li deposition with P(VDF‐TrFE) and PTZN electrolytes. e) XPS analysis of Li metal surface after cycling with P(VDF‐TrFE) and PTZN electrolytes.

In order to investigate the effect of ZIF‐90‐NH_2_ filler on Li metal anodes, we assembled Li || Li symmetric cells with different solid electrolytes and tested their Li plating/stripping behavior. As shown in **Figure** **5**a, across a range of current densities from 0.1 to 0.5 mA cm^−2^, the Li/PTZN/Li cells consistently exhibited lower polarization voltages compared to the Li/P(VDF‐TrFE)/Li cells. In particular, when the current density was increased to 0.5 mA cm^−2^, the overpotential of Li/P(VDF‐TrFE)/Li cell increased until a short circuit occurred, due to the unstable electrode/electrolyte interface. On the contrary, Li/PTZN/Li cell maintained stable cycling with a polarization voltage of 80 mV, showcasing excellent rate performance by sustaining a minimal polarization voltage of 21 mV when the current density returned to 0.1 mA cm^−2^. Under the 0.2 mA cm^−2^/0.2 mAh cm^−2^, the polarization voltage of Li/P(VDF‐TrFE)/Li cells began to rise after only 460 h of cycling, ultimately leading to a short circuit, whereas Li/PTZN/Li cells can be stably cycled for 1000 h and maintain a low polarization voltage of 30 mV, which can be attributed to the high Li^+^ transference number and stable SEI layer on the anode surface (Figure 5b). Meanwhile, the voltage plateau of the Li/PTZN/Li cell was smoother than that of the Li/P(VDF‐TrFE)/Li cell during cycling, indicating that the incorporation of ZIF‐90‐NH_2_ brings a more homogeneous and stable Li deposition. In addition, the critical deposition capacity (CDC) of Li/PTZN/Li cells was 1.4 mAh cm^−2^, demonstrating that the PTZN electrolyte can match cathode materials with high loading (Figure S14, Supporting Information).^[^ ^31^ ^]^

The surface morphology of the Li metal anode after cycling showed that the Li anode surface of Li/P(VDF‐TrFE)/Li cell displayed a large number of rough Li dendrites after only 30 cycles. On the contrary, after cycling with PTZN electrolyte, the Li anode surface remained flat and smooth without any Li dendrites (Figure 5c). Simultaneously, the P(VDF‐TrFE) electrolyte's surface became rough, with lithium dendrites adhering to it after cycling, whereas the PTZN electrolyte maintained a smooth and intact structure (Figure S15, Supporting Information). Besides, the interfacial impedance of the P(VDF‐TrFE) electrolyte underwent significant changes before and after cycling, while the PTZN electrolyte's interfacial impedance remained nearly constant, demonstrating excellent interfacial stability (Figure S16, Supporting Information). It is mainly because the polymer chains in the P(VDF‐TrFE) electrolyte are entangled with each other in a haphazard arrangement, and the free TFSI^−^ anions restrict Li^+^ transport, leading to concentration polarization and uneven electric field distribution at the electrode surface, which results in uneven Li deposition and Li dendrites (Figure 5d). In contrast, after introducing ZIF‐90‐NH_2_, the integrating competitive Li^+^ coordination with immobilized anions in composite solid electrolyte promotes the orderly arrangement of polymer chains, while the Lewis acidic metal sites of ZIF‐90‐NH_2_ and the positively charged groups of ‐NH_2_ firmly adsorb the TFSI^−^ anions, which greatly enhances the Li^+^ transport ability and brings about uniform Li deposition.

Furthermore, the SEI layer on Li metal surface after cycling was analyzed by X‐ray photoelectron spectroscopy (XPS). As shown in Figure 5e, the peaks corresponding to C─C/H, C ═ O, and LiN_3_ in the SEI layer using P(VDF‐TrFE) electrolyte were much larger than those using the PTZN electrolyte, which can be attributed to the decomposition of DMF, highlighting the strong adsorption of DMF molecules with ZIF‐90‐NH_2_. Concurrently, the Li 1s and F 1s spectra indicated that the predominant component of the SEI layer was LiF, with its content being higher in the SEI layer of the PTZN electrolyte compared to the P(VDF‐TrFE) electrolyte, suggesting that more Li salts were dissociated from the PTZN electrolyte. EDS results further confirmed that LiF was uniformly distributed within the SEI layer when using PTZN electrolyte (Figure S17, Supporting Information). The presence of LiF aids in promoting uniform Li deposition and inhibiting dendritic Li growth.^[^ ^32^ ^]^ Notably, the H─C─F peak in the C 1s spectra corresponded to the decomposition of P(VDF‐TrFE), indicating a strong interfacial adhesion of the PTZN electrolyte to the Li metal anode.^[^ ^33^ ^]^ On the contrary, the P(VDF‐TrFE) electrolyte exhibited a porous, rough surface with poor interfacial contact with the Li anode, resulting in the absence of the H─C─F peak in the SEI layer. These XPS results demonstrated that ZIF‐90‐NH_2_ can effectively immobilize TFSI^−^ anions and DMF molecules, thereby weakening the binding strength of Li^+^‐O in the solvation structure of [Li(DMF)_x_]^+^ and regulating the Li^+^ competitive coordination, thus inhibiting the decomposition of DMF at the interface and further forming stable SEI layer. Moreover, Tafel curves showed that the exchange current density of Li/PTZN/Li cell was 0.29 mA cm^−2^, surpassing that of the Li/P(VDF‐TrFE)/Li cell (0.22 mA cm^−2^), indicating that the SEI layer had a faster Li^+^ transport kinetics (Figure S18, Supporting Information).

### 2.4 Full Cells and Application

**Figure 6:**
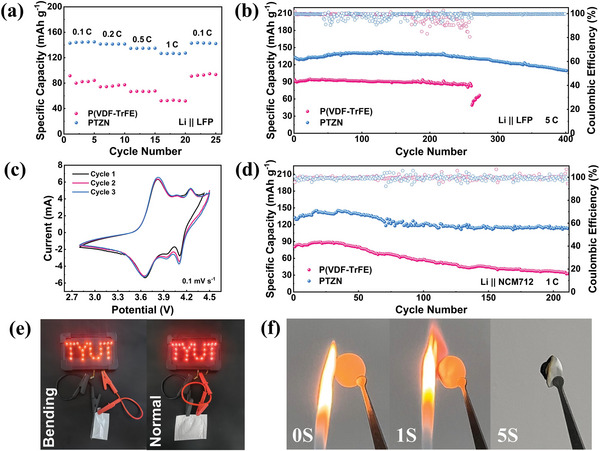
a) Rate performance and b) long‐cycle performance of Li || LFP cells with P(VDF‐TrFE) and PTZN electrolytes at room temperature. c) CV curves and d) long‐cycle performance of Li || NCM712 cells with P(VDF‐TrFE) and PTZN electrolytes. e) Pouch cells under normal and folded for LED light panels. f) Flame retardancy test of PTZN electrolyte.

Full cells with LiFePO_4_ (LFP) cathode and LiNi_0.7_Co_0.1_Mn_0.2_O_2_ (NCM712) cathode were assembled to test the advancement of PTZN electrolyte in SSBs at room temperature. As shown in **Figure** **6**a, the discharge‐specific capacities of the Li/PTZN/LFP cells were 144, 141, 134, and 126 mAh g^−1^ at 0.1 C, 0.2 C, 0.5 C and 1 C, respectively. When the rate was returned to 0.1 C, its discharge‐specific capacity recovered to 143 mAh g^−1^, showing a good rate performance. On the contrary, the Li/P(VDF‐TrFE)/LFP cells demonstrated low discharge‐specific capacity at each rate.^[^ ^5a^ ^]^ In addition, the long‐cycle performance of Li || LFP cells at 1 C and 5 C was tested. As shown in Figure S19 (Supporting Information), the initial discharge‐specific capacity of the Li/PTZN/LFP cells at 1 C was 155 mAh g^−1^, and it can maintain a high capacity retention rate of 99% after 200 cycles. In contrast, the capacity of the Li/P(VDF‐TrFE)/LFP cells began to decline markedly after 30 cycles, with a capacity retention of only 23% after 200 cycles. Remarkably, when the rate was increased to 5 C, the initial discharge‐specific capacity of the Li/PTZN/LFP cells was still as high as 133 mAh g^−1^, and it can run stably for more than 400 cycles with a capacity retention of 82%, demonstrating high cycling stability. However, when using P(VDF‐TrFE) electrolytes, the cells displayed a low capacity at 5 C and short‐circuits after 260 cycles (Figure 6b). The charge‐discharge curves showed that the Li/PTZN/LFP cells maintained a small overpotential throughout 200 cycles (Figure S20a, Supporting Information). The cyclic voltammetry (CV) curves showed that there were not any side reactions, underscoring excellent redox reversibility (Figure S21a, Supporting Information). This fact can be attributed to the immobilization of residual DMF molecules by ZIF‐90‐NH_2_, preventing side reactions with Li metal.

Similarly, the Li || NCM712 cells were also assembled to test the stability of CSEs at high voltage (4.5 V). As shown in Figure 6d, the initial discharge‐specific capacity of the Li/PTZN/NCM712 cells was 132 mAh g^−1^ at 1 C, with a capacity retention of 85% after 200 cycles, showcasing excellent high‐voltage stability. This performance is attributed to the hydrogen bonding within the PTZN electrolyte, which enhances antioxidant properties, while its high ionic conductivity and Li^+^ transference number contribute to robust interface stability. In stark contrast, the Li/P(VDF‐TrFE)/NCM712 cells only exhibited an initial specific capacity of 80 mAh g^−1^, with rapid capacity decay after just 40 cycles, reaching a retention rate of 45% at 200 cycles. The charge‐discharge curves demonstrated that the Li/PTZN/NCM712 cells maintained a small polarization voltage throughout 200 cycles (Figure S20b, Supporting Information). Meanwhile, the CV curves confirmed that no peaks of side reactions appeared at different scan rates of 0.1 and 0.2 mV s^−1^, exhibiting good redox reversibility (Figure 6c; Figure S21b, Supporting Information). All results show that the PTZN electrolyte is well‐suited for the high‐voltage NCM712 cathode. Furthermore, to verify the safety of the PTZN electrolyte, a flame retardancy test was conducted. As shown in Figure 6f, the PTZN electrolyte ceased to burn within 5 s of flame exposure, indicating commendable thermal stability. Additionally, the assembled pouch cells can still light up the light panel during the folding process, showing good safety and application feasibility (Figure 6e).

## 3 Conclusion

In summary, we designed a composite solid electrolyte based on integrating competitive Li^+^ coordination with immobilized anions to address the persistent challenges of low ionic conductivity and undesirable decomposition of DMF solvents in PVDF‐based SPEs. Specifically, the CSEs were constructed by combining P(VDF‐TrFE) in its all‐trans conformation with ZIF‐90‐NH_2_. Through the synergistic effects of Lewis acid‐base interactions and electrostatic forces, the functionalized ZIF‐90‐NH_2_ can effectively immobilize DMF molecules and TFSI^−^ anions, inhibiting the decomposition of DMF at the interface and inducing the rearrangement of Li^+^ solvation structure, thus improving interface stability and achieving high ionic conductivity. The P(VDF‐TrFE) matrix, with its all‐trans conformation, aligns F atoms uniformly on the carbon chain, meanwhile, hydrogen bonding interactions enhance the electrochemical window of CSEs and facilitating an ordered arrangement of ZIF‐90‐NH₂ with polymer chains, accelerating the Li^+^ transference. Consequently, the full cells with LFP cathode can be stably cycled more than 400 cycles with 82% capacity retention at a high rate of 5 C. The Li || NCM712 cells can also be stably cycled more than 200 cycles at a high cut‐off voltage of 4.5 V, demonstrating excellent high‐voltage stability. This study provides a new way to design CSEs with high ionic conductivity and interfacial stability.

## Conflict of Interest

The authors declare no conflict of interest.

## Supporting information



Supporting Information

## Data Availability

The data that support the findings of this study are available from the corresponding author upon reasonable request.
