# Supplementary material for: Integrating Competitive Li+ Coordination with Immobilized Anions in Composite Solid Electrolyte for High‐Performance Li Metal Batteries
Source: Adv Sci (Weinh). 2025 Feb 18;12(14):2413875. doi: 10.1002/advs.202413875 (PMC11984912; doi:10.1002/advs.202413875)
Supplement: Supplementary file 1 — Supporting Information [file ADVS-12-2413875-s001.docx]

**Supporting Information**

**Integrating competitive** **Li^+^ coordination with** **immobilized anions in composite solid electrolyte for** **high-performance Li metal batteries**

*Ziyang Liang, Chang Liu, Xiang Bai, Jiahui Zhang, Xinyue Chang, Bo Zhang, Mengxue Xia, Huayun Du, Hao Huang*, Bing Wu*, Chengkai Yang*, Shi Wang*, Wen Liu* *and Qian Wang**

1. **Experimental Section**
   1. **Preparation of ZIF-90**

Zinc acetate dihydrate (274 mg) was added to a mixed solution containing equal amounts of deionized water (10 mL) and tert-butanol (10 mL), and stirred to dissolve to obtain the trigger solution A. Imidazole-2-carboxaldehyde (480 mg) and polyvinylpyrrolidone (50 mg) were added to a mixed solution containing deionized water (15 mL) and glycerol (5 mL), and the solution was stirred at 60 ℃ until the solution was clarified to obtain solution B. Solution A and B were mixed and stirred vigorously at room temperature for 15 min. Finally, the resulting product was collected by centrifugation (8000 g), washed three times with excess ethanol and dried under vacuum at 60 ℃.

- 1. **Preparation of ZIF-90-NH_2_**

The ZIF-90 powder (150 mg) obtained above was added to a mixed solution containing 0.05 mL of ethylenediamine and 40 mL of methanol, and the reaction was stirred at 60 ℃ for 24 h. The resultant product was collected by centrifugation (8000 g), washed with excess methanol and dried under vacuum at 60 ℃ for 12 h.

- 1. **Preparation of PTZN electrolytes**

The P(VDF-TrFE) precursor solution was obtained by adding 0.3 g of P(VDF-TrFE) and 0.21 g of LiTFSI to 6 mL of DMF solution and stirring for 6 h at room temperature. After that, ZIF-90-NH_2_ powder was weighed at a mass ratio of [P(VDF-TrFE) + LiTFSI]:ZIF-90-NH_2_ = 100:1. The powder was added to the P(VDF-TrFE) precursor solution and stirred at room temperature for 12 h until no precipitation was observed to obtain the PTZN solution. Finally, 100 μL of PTZN solution was added dropwise to the cellulose diaphragm and dried in a glove box for 24 h to obtain the PTZN electrolyte.

- 1. **Sample characterization**

Transmission electron microscopy (TEM, Japan Electron JEM 2010, Japan) and scanning electron microscopy (SEM, Hitachi S4800, Japan) were used to characterize the morphology and structure of the samples. X-ray diffraction (XRD) characterization was performed under a Cu Kα light source. Fourier transform infrared (FTIR) spectra of the samples were obtained by Nicolet iS50 spectrometer. The specific surface area and pore size of the samples were tested using a fully automated specific surface and porosity analyzer (Micromeritics ASAP 2460, USA). Young's modulus and surface roughness of the samples were obtained by atomic force microscopy (AFM, Bruker Dimension Icon, Germany). The ^19^F nuclear magnetic resonance (NMR) spectra of the samples were obtained by Bruker 500MHz. Differential scanning calorimetry (DSC) analyses were tested using a Netzsch DSC 200 F3 instrument with a temperature range of -100 to 120 °C and a ramp rate of 10 °C min^-1^. Thermogravimetric analyses (TGA) were performed by ramping the sample from 30 °C to 500 °C at a rate of 10 °C min^-1^ under nitrogen atmosphere. The instrument used was a Netzsch STA449 thermal analyzer. The composition of the SEI layer on the lithium anode surface was analyzed by X-ray photoelectron spectroscopy (XPS, Thermo Scientific K-Alpha, USA).

- 1. **Electrochemical measurements**

Ionic conductivity is measured in a Metrohm PGSTAT302N electrochemical workstation. The frequency range was from 0.1 Hz to 1 MHz and the temperature range was from 30 to 80 °C. The electrolyte was sandwiched between two stainless steel sheets (SS) and assembled into an SS/electrolyte film/SS cell for measurement. The ionic conductivity (σ) was calculated by the following Equation (1):

$\sigma=\frac{L}{RS}$ (1)

Where R is the intrinsic impedance, L is the thickness of the electrolyte, and S is the area of the electrolyte in contact with the stainless steel sheet.

Li^+^ transference number (t_Li+_) was measured using a Li/electrolyte film/Li symmetric cell in a Metrohm PGSTAT302N electrochemical workstation. A polarization voltage of 10 mV was applied to the cell to obtain the current time curve and AC impedance spectrum. The t_Li_^+^ was calculated by the following Equation (2):

$t_{{Li}^{+}}=\frac{I_{s}(\Delta V-R_{0}I_{0})}{I_{0}(\Delta V-R_{s}I_{s})}$ (2)

Where ∆V is the polarization voltage, I_0_ is the initial current of polarization, Is is the polarization steady state current, R_0_ is the interfacial impedance before polarization and R_s_ is the interfacial impedance after polarization.

SS || Li cells were tested using linear sweep voltammetry (LSV). The voltage range was from 2 to 6 V with a scan rate of 5 mV s^-1^. Cyclic voltammetry (CV) was used to test the Li || LFP and Li || NCM712 cells. The voltage ranges were 2.5 to 4.2 V and 2.8 to 4.5 V with scan rates of 0.1 and 0.2 mV s^-1^, respectively.

- 1. **Density functional theory calculations**

The density functional theory (DFT) calculation were performed with the CP2K quantum chemistry software package,^[1]^ using Perdew–Burke–Ernzerhof (PBE) parametrization of generalized gradient approximation (GGA) to describe the exchange-correlation part in Hamiltonian. We use GTH potential and Molopt basis set (DZVP-MOLOPT-SR-GTH) with an energy cutoff of 400 Ry.^[2]^ Van der Waals (vdW) interaction was taken into account at the DFT-D3 level as proposed by Grimme.^[3]^ Both atomic position and cell parameters were relaxed until the max force is lower than 4.5E-4 Ha/bohr. All calculation were spin-polarized.

Calculation of adsorption energy (*E_ads_*)

$E_{ads}=E(A+B)-E(A)-E(B)$ (3)

1. **Supplementary figures**


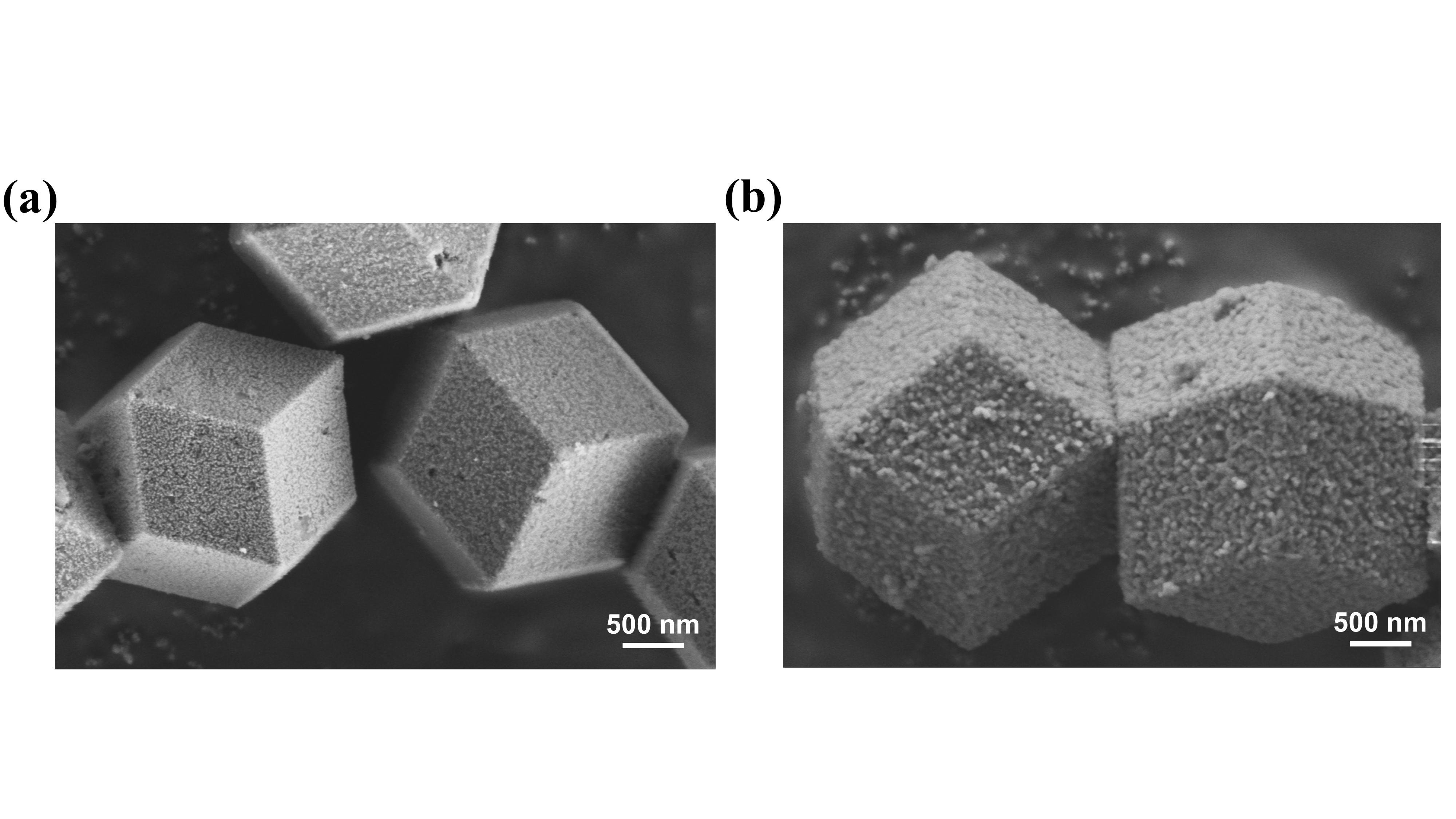


**Figure S1.** SEM images of ZIF-90 (a) and ZIF-90-NH_2_ (b).


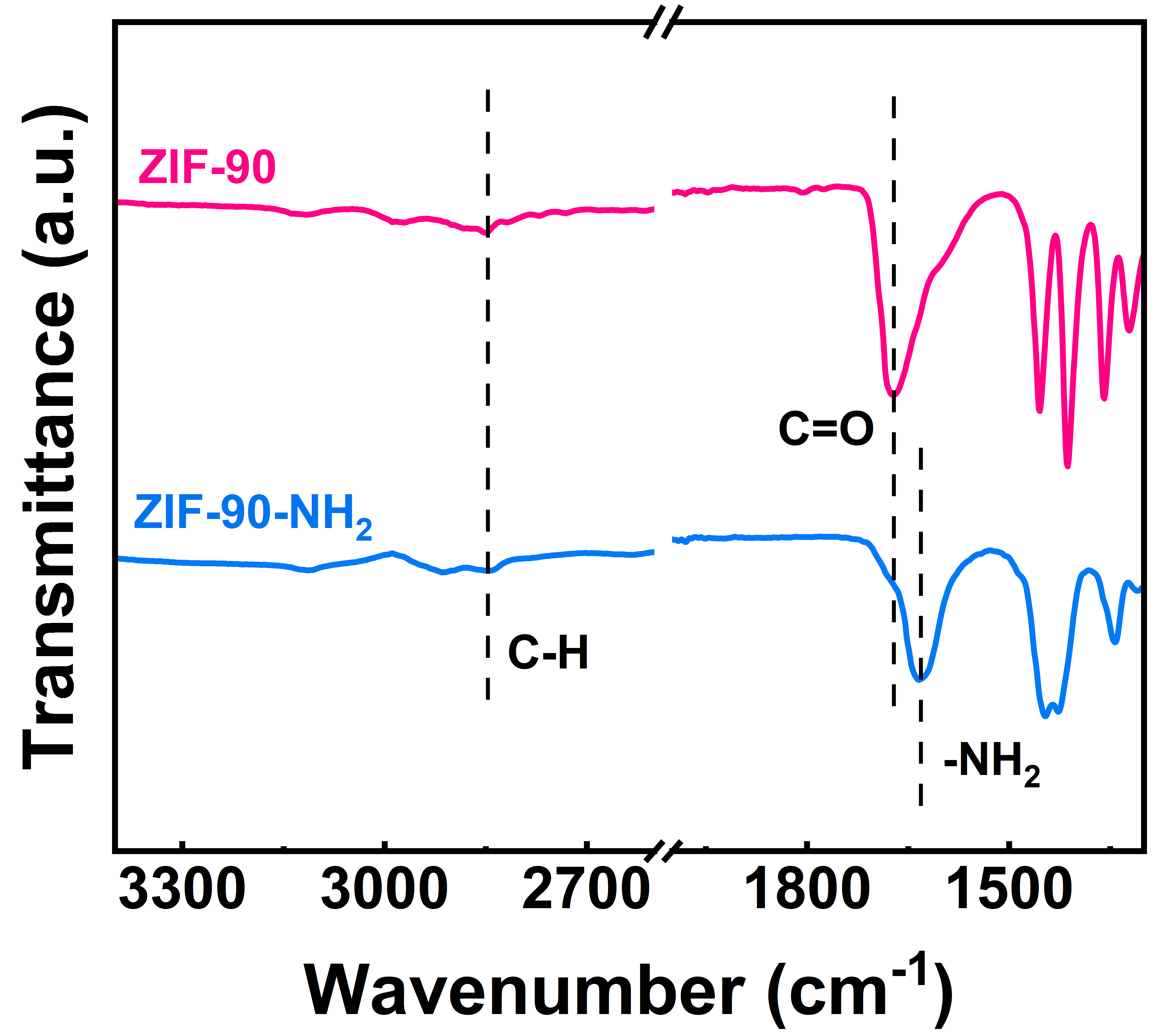


**Figure S2.** FTIR spectra comparison of ZIF-90 and ZIF-90-NH_2_.


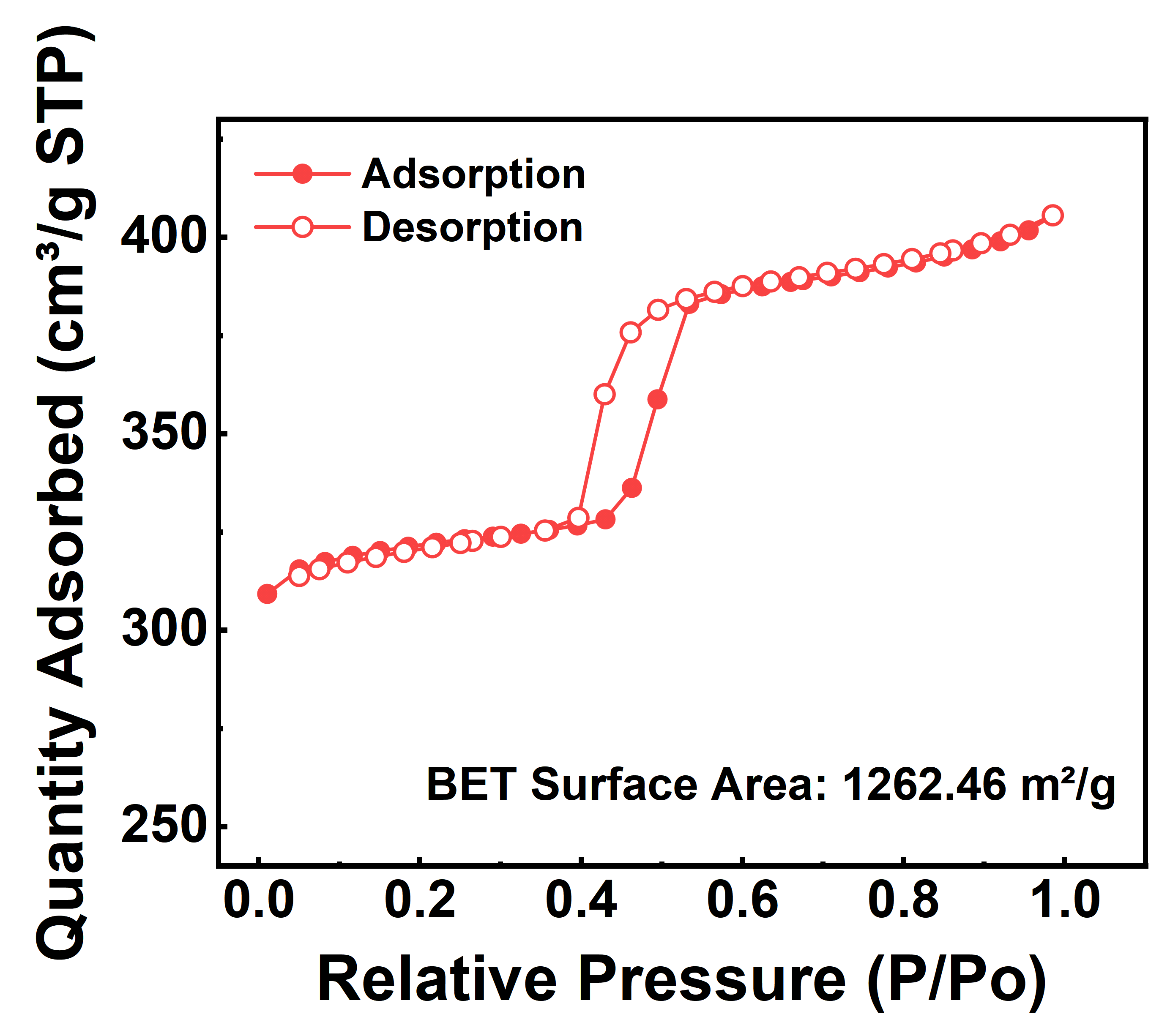


**Figure S3.** The BET specific surface area of ZIF-90-NH_2_ .


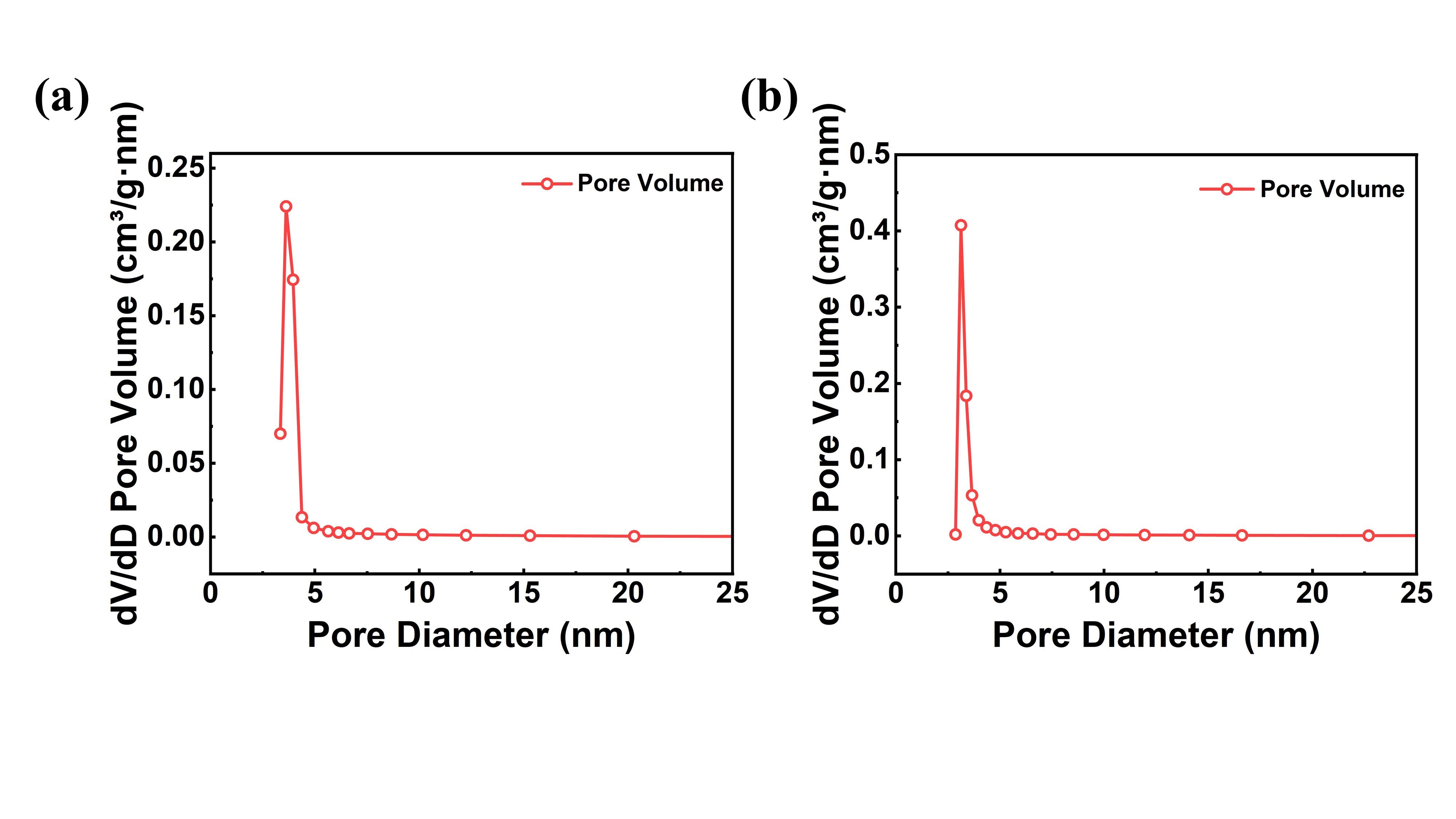


**Figure S4.** Nitrogen adsorption (a) and desorption (b) isotherms of ZIF-90-NH_2_ .


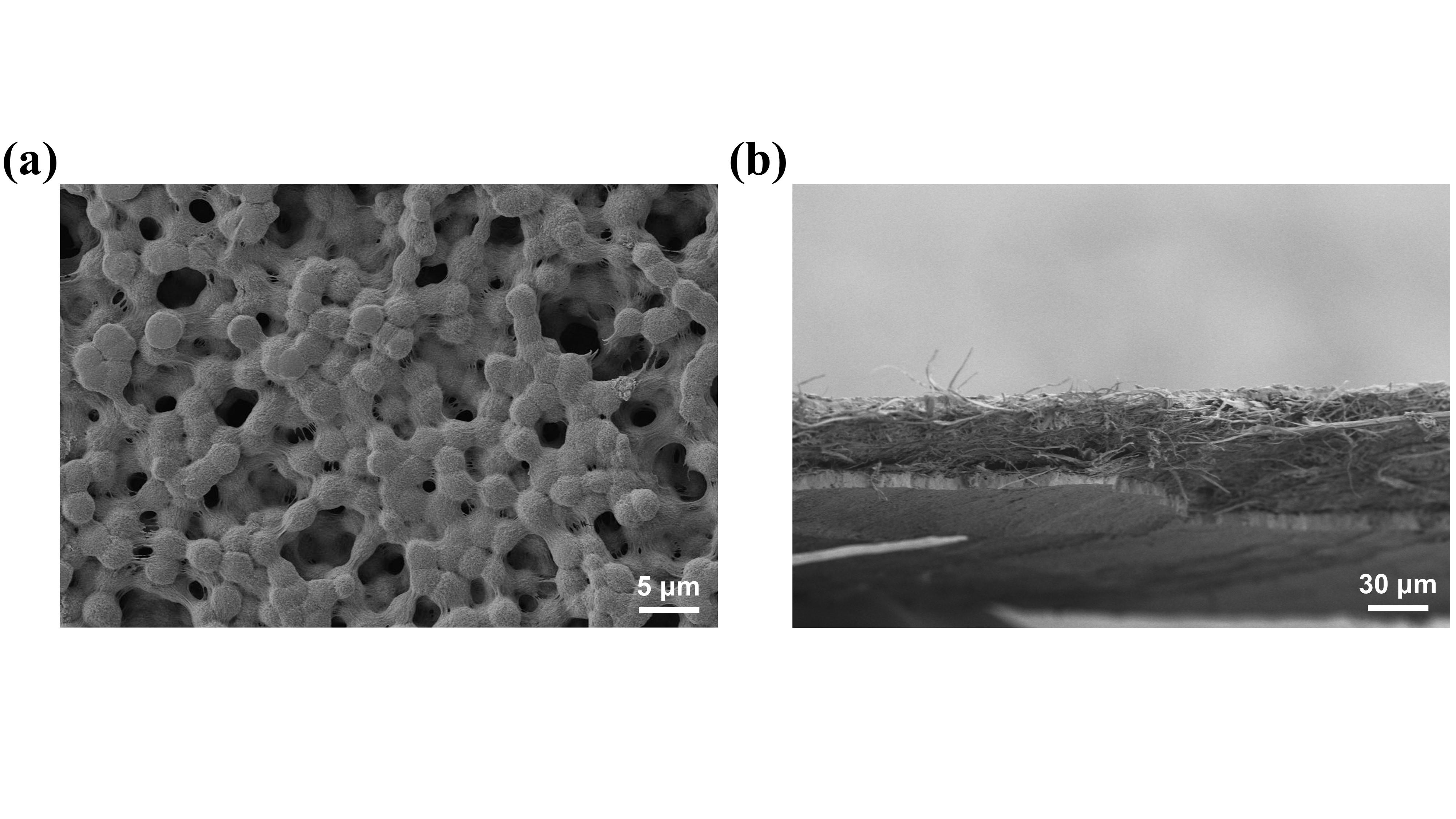


**Figure S5.** Surface (a) and cross-sectional (b) SEM images of P(VDF-TrFE) electrolyte.


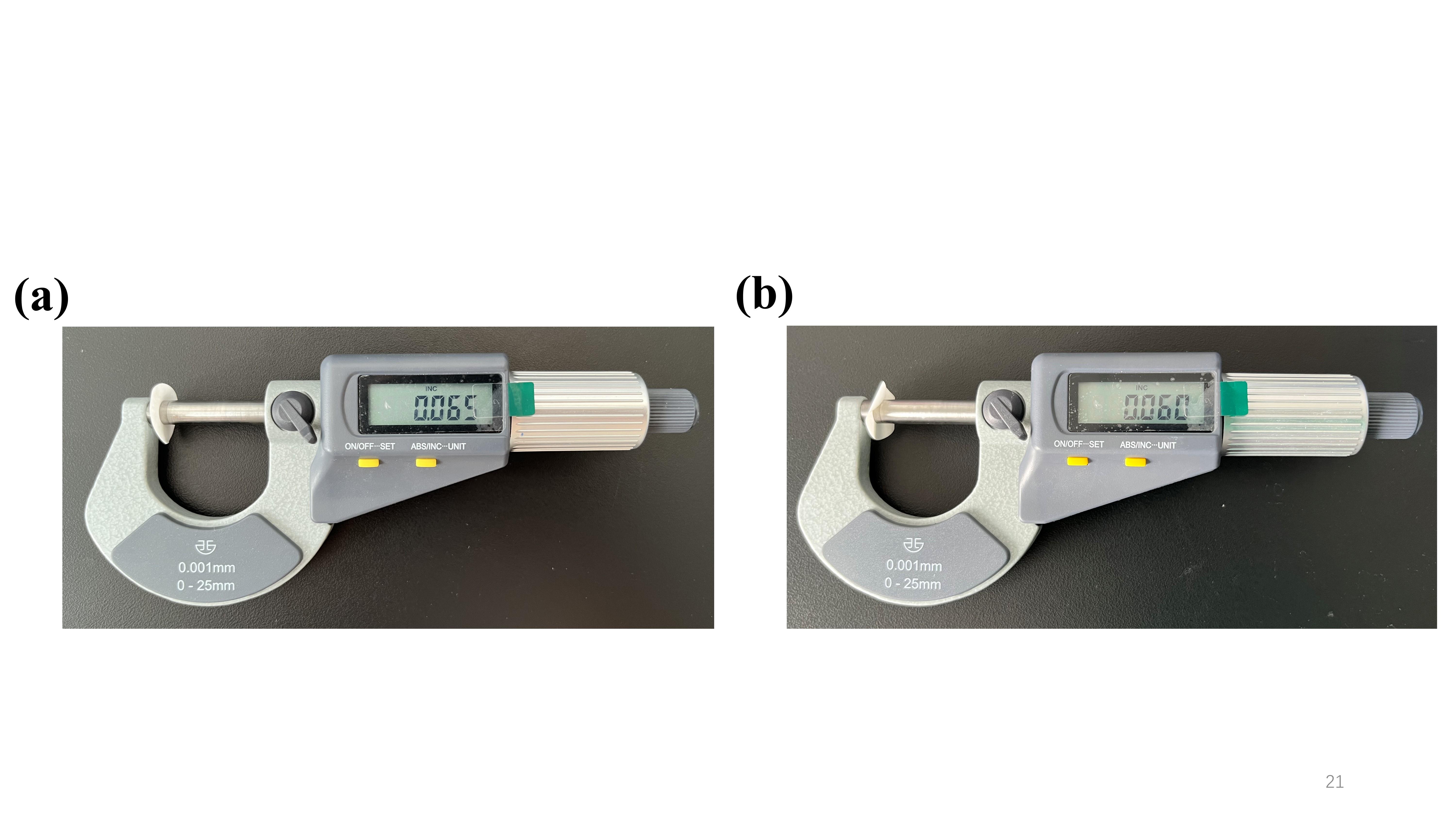


**Figure S6.** Thickness measurement of P(VDF-TrFE) electrolyte (a) and PTZN electrolyte (b) film.


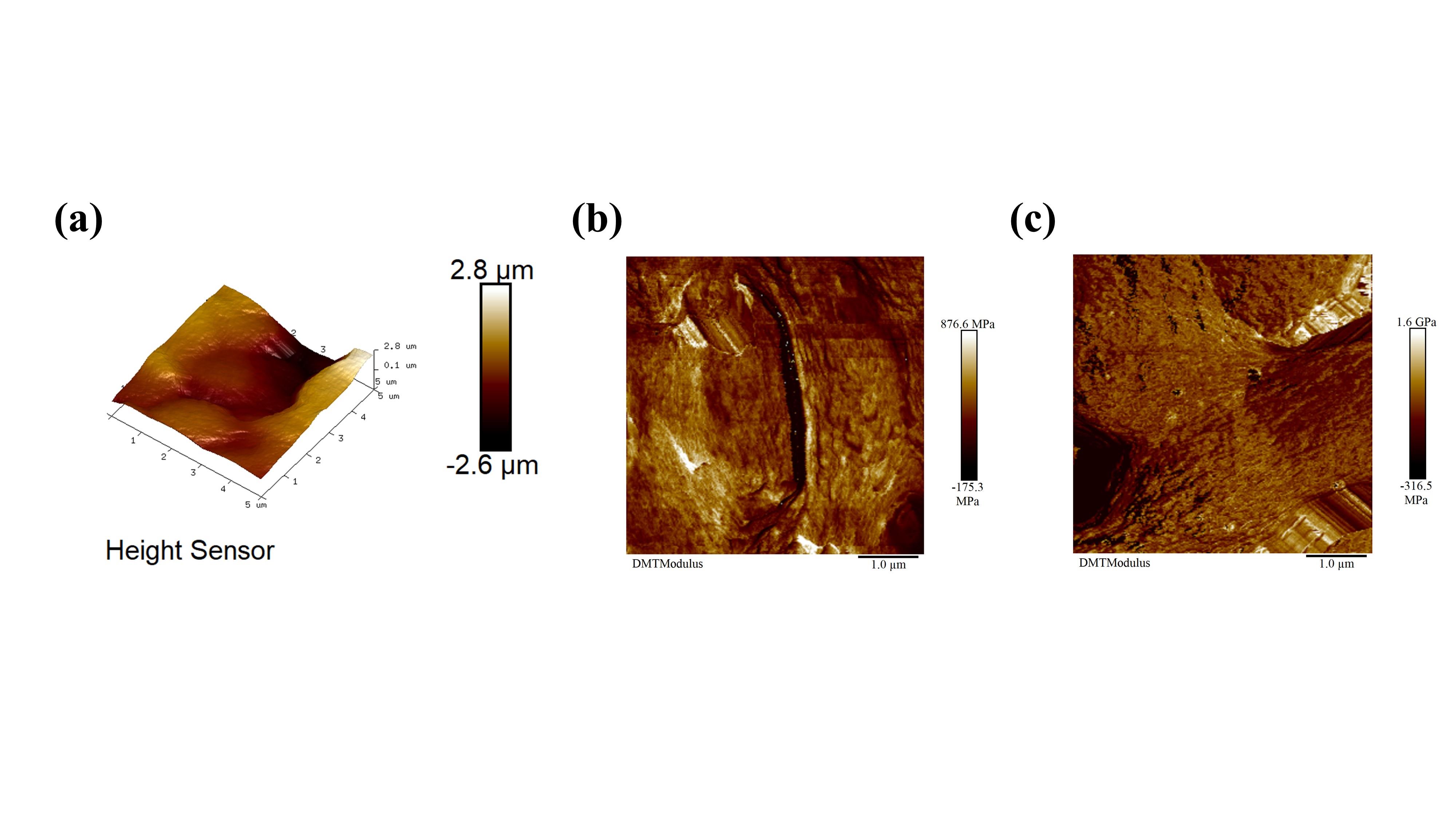


**Figure S7.** (a) Surface roughness of P(VDF-TrFE) electrolyte. Young's modulus of P(VDF-TrFE) (b) and PTZN (c) electrolytes.


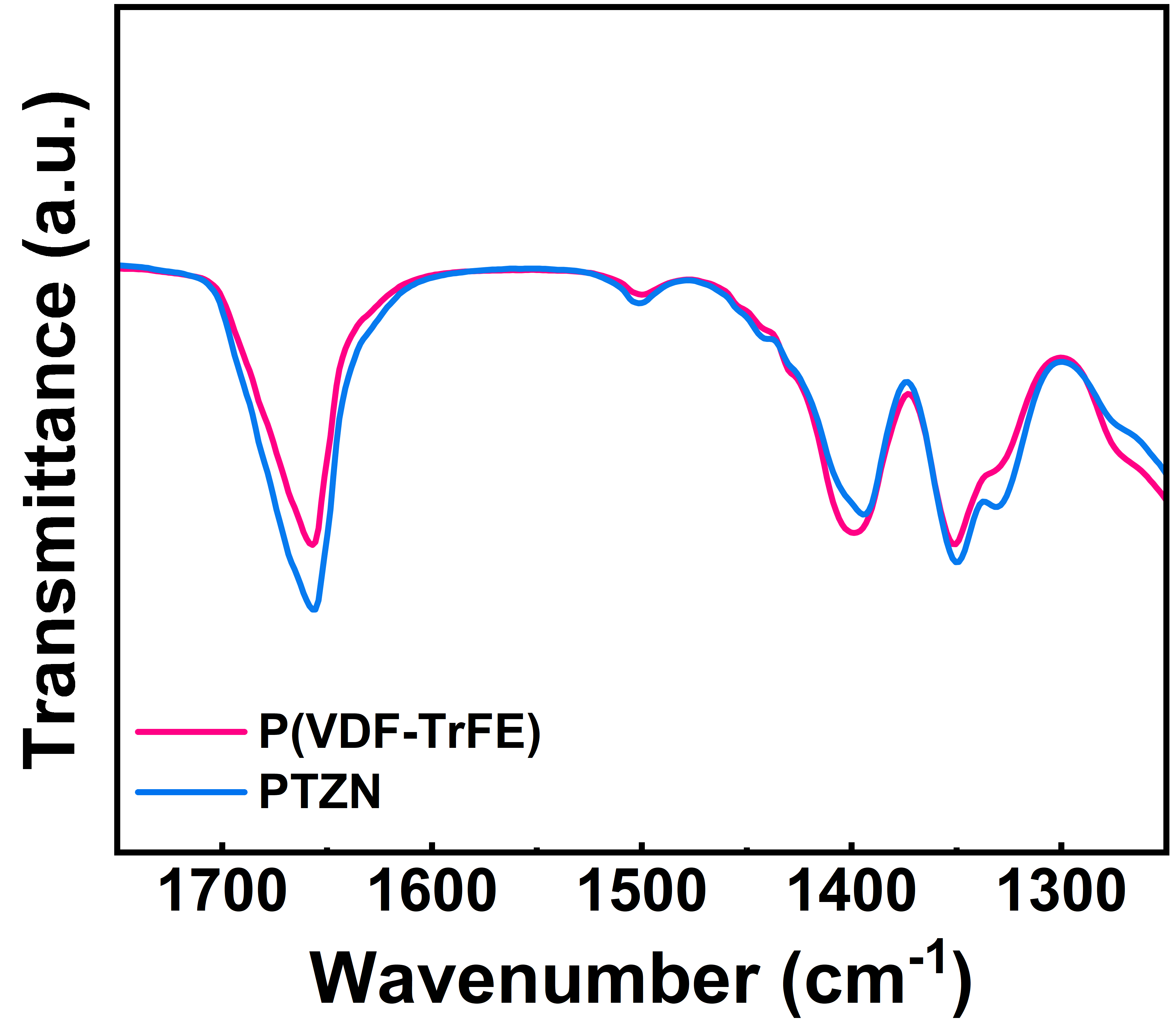


**Figure S8.** FTIR spectra of P(VDF-TrFE) and PTZN electrolytes in different ranges.


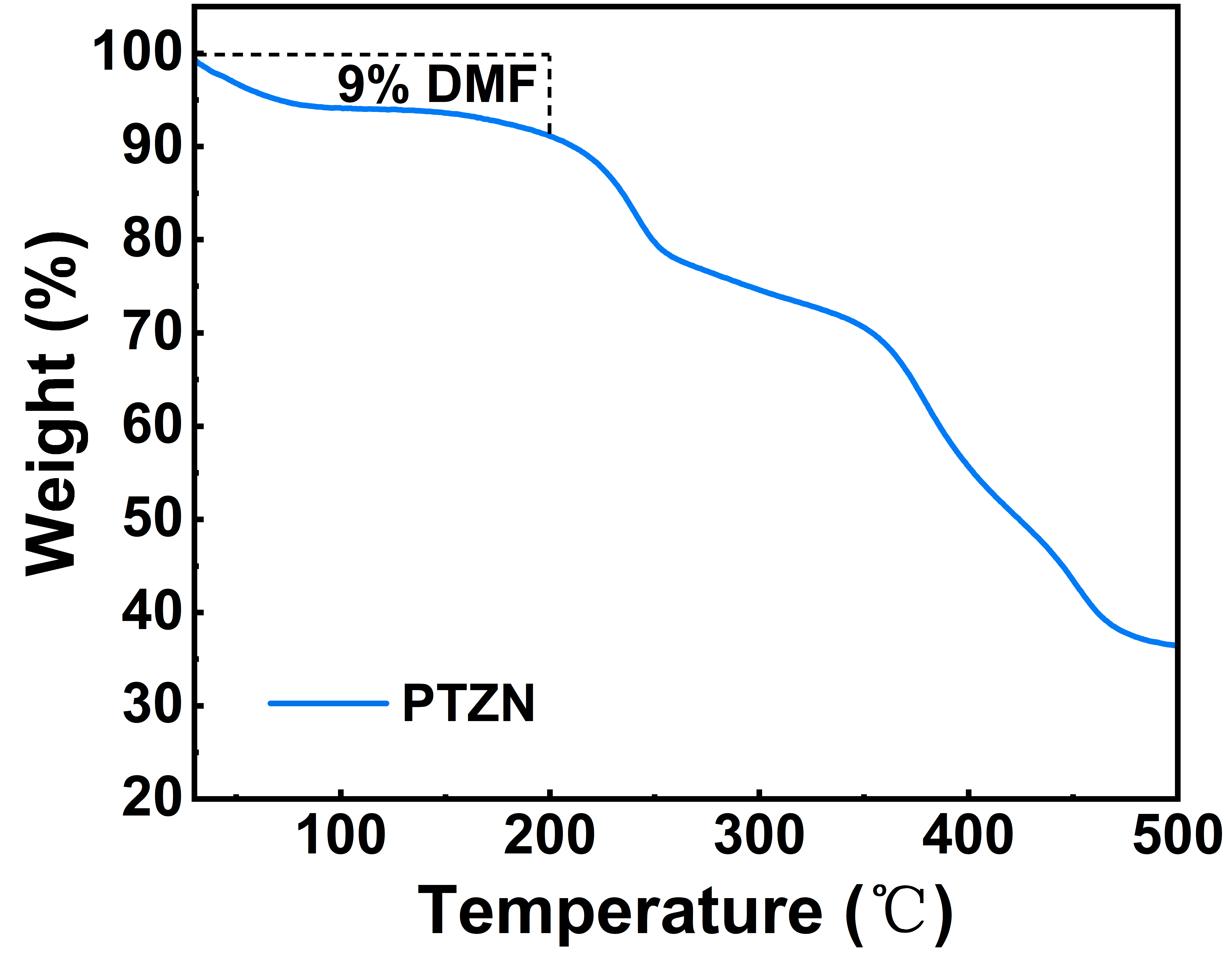


**Figure S9.** TGA curves of PTZN electrolytes after cycling.


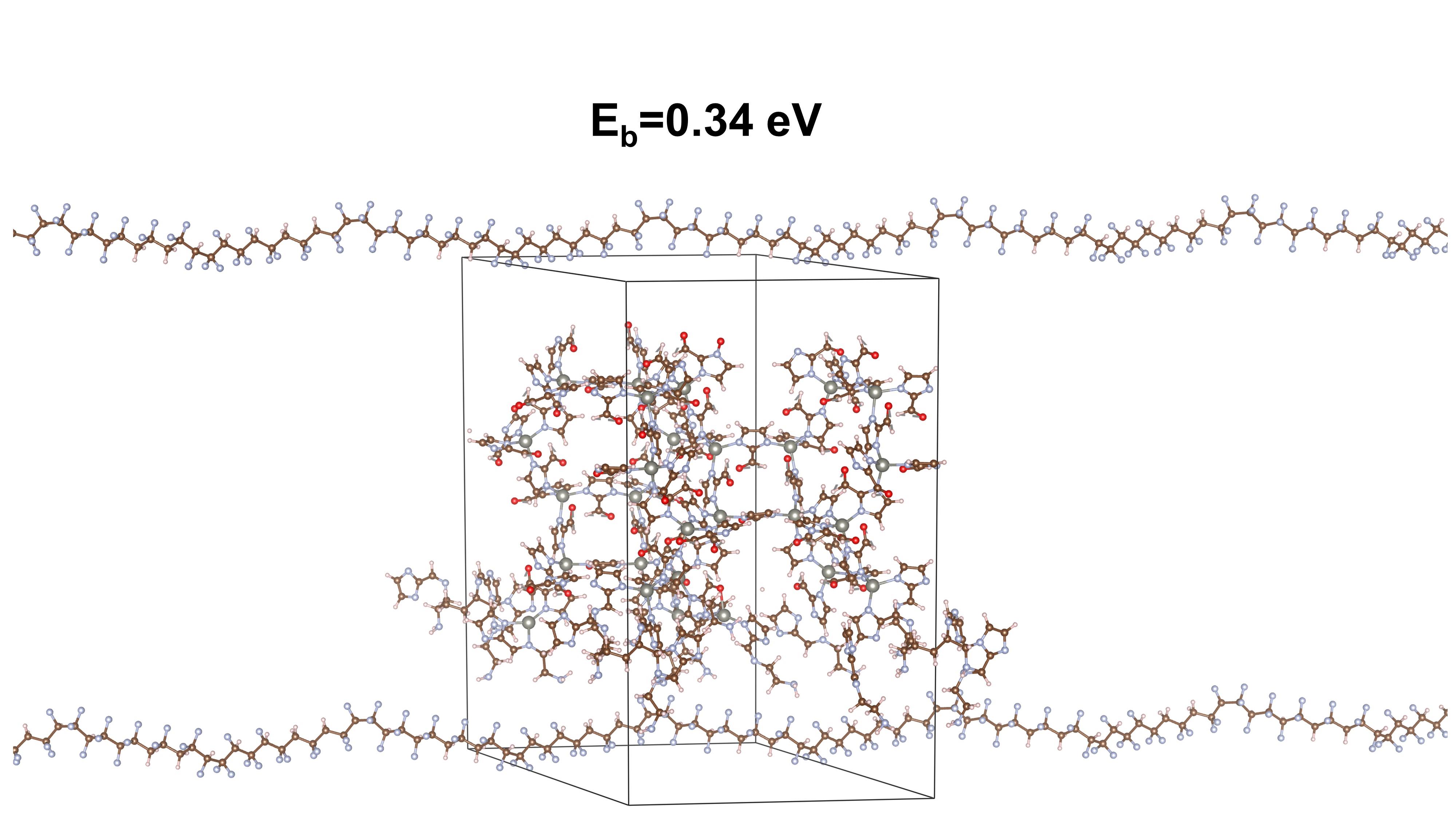


**Figure S10.** Binding energy of P(VDF-TrFE) to ZIF-90-NH_2_.


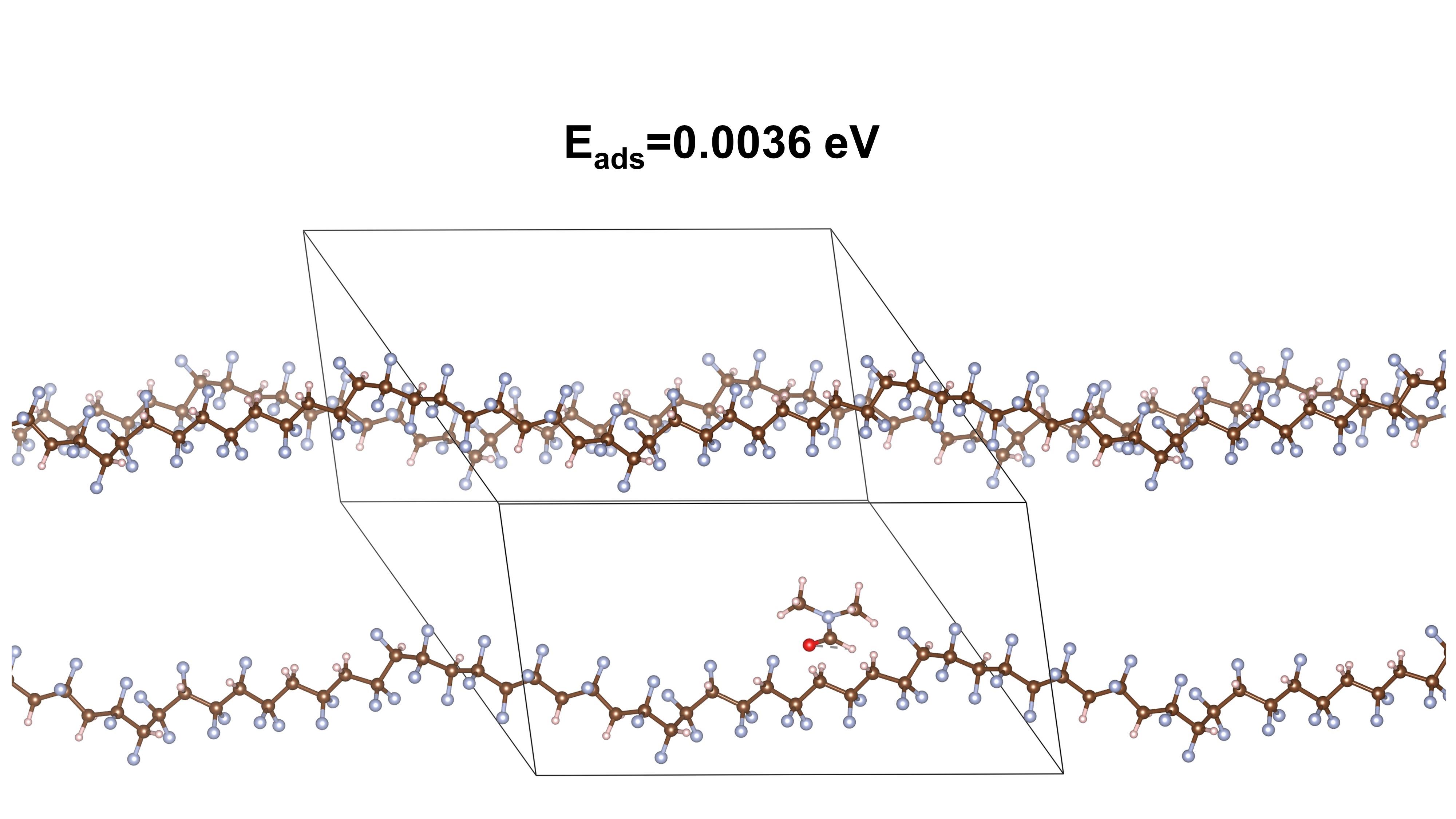


**Figure S11.** Adsorption energy of DMF molecules on P(VDF-TrFE).


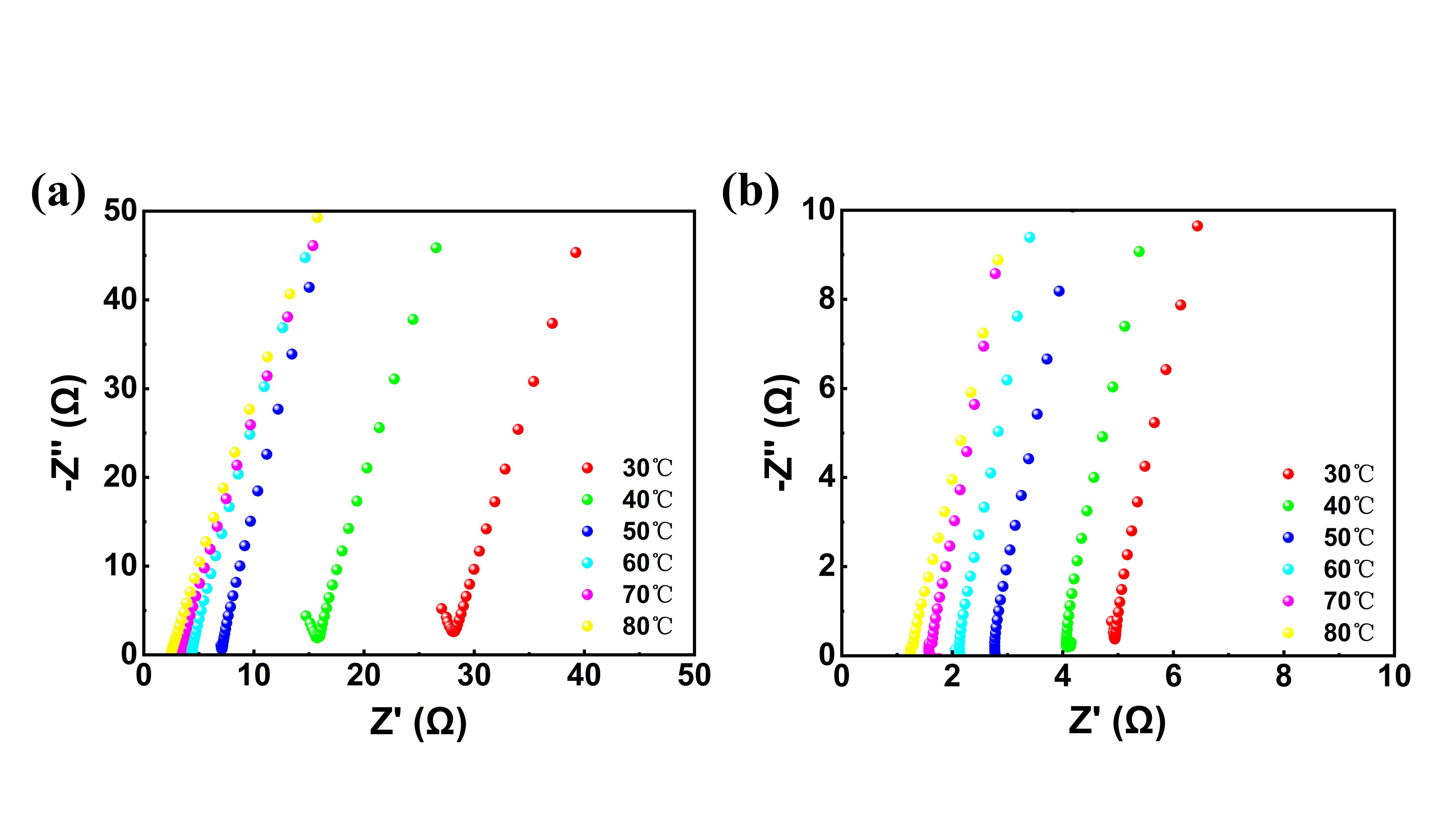


**Figure S12.** Nyquist plots of P(VDF-TrFE) electrolyte (a) and PTZN electrolyte (b) at different temperatures.


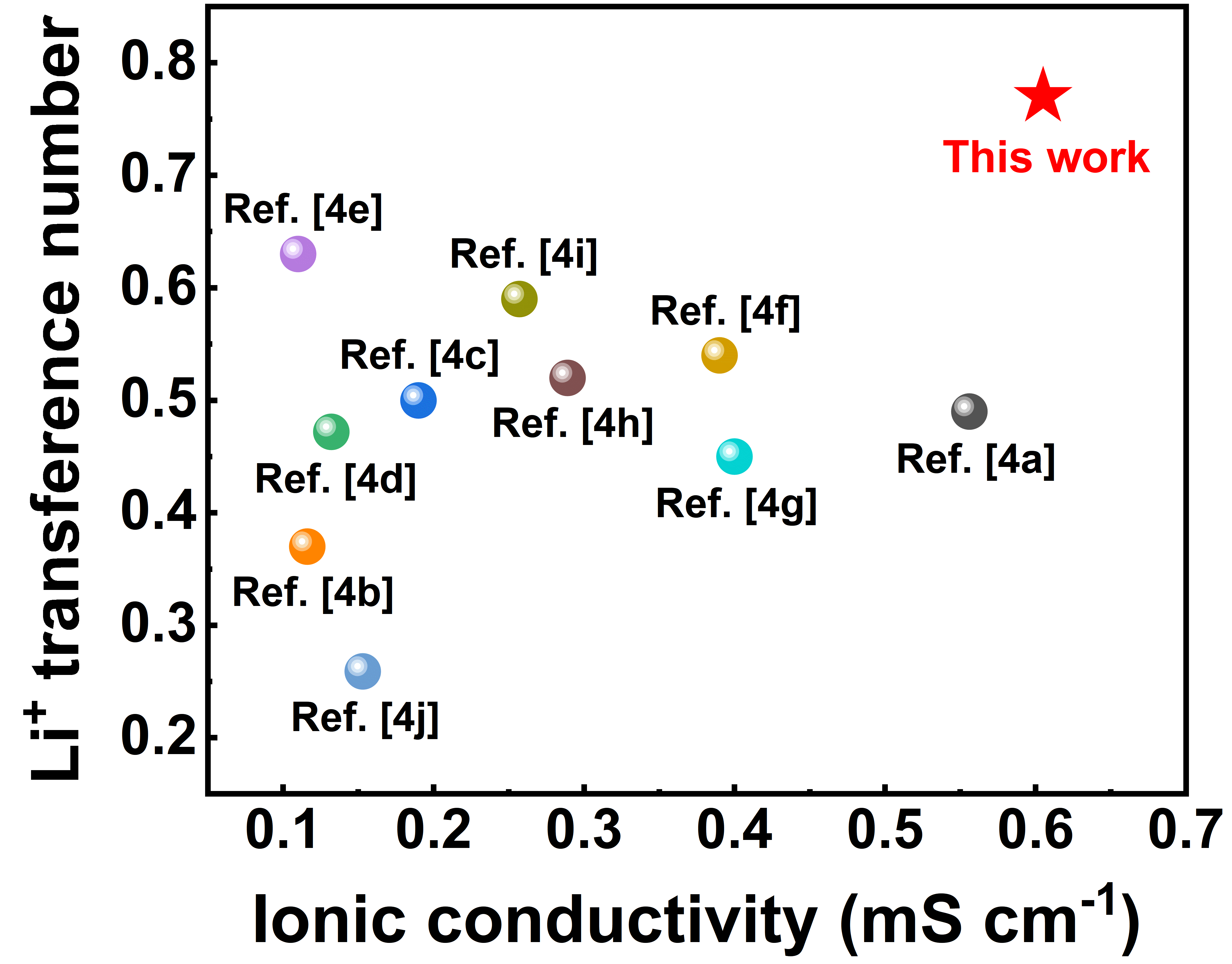


**Figure S13.** Comparison of various PVDF-based solid-state electrolytes.^[4]^


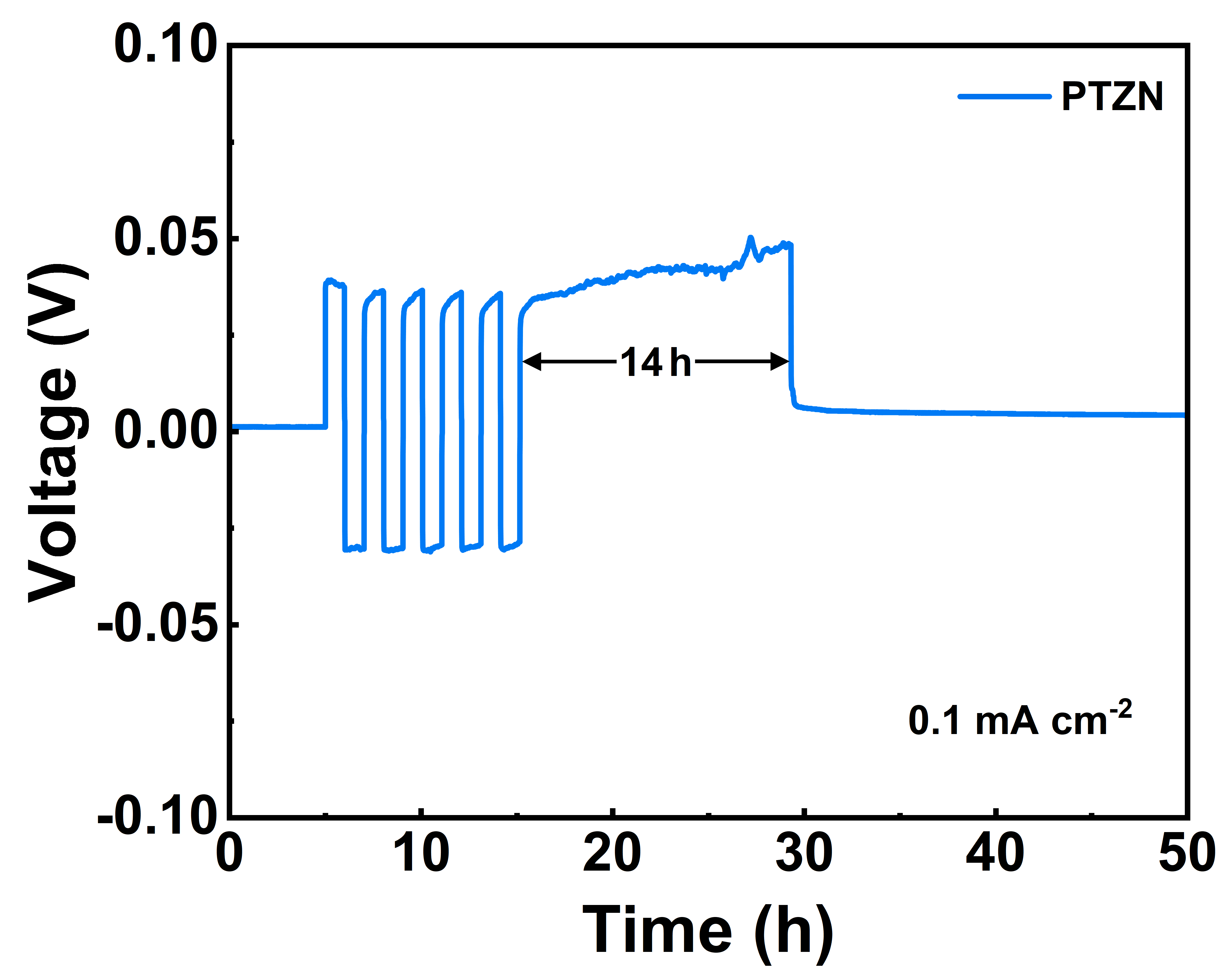


**Figure S14.** CDC test of Li/PTZN/Li cell.


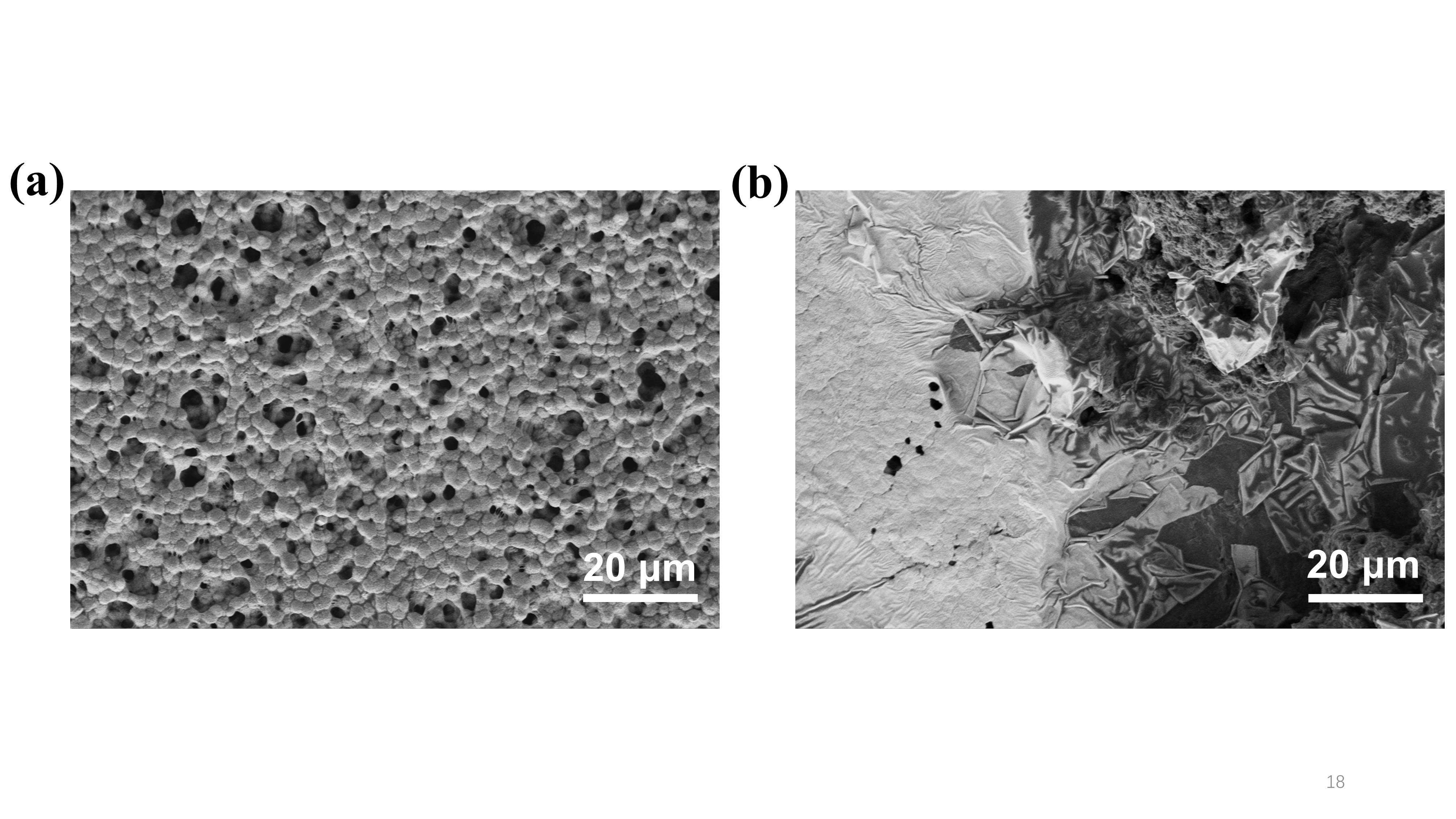


**Figure S15.** Surface SEM images of PTZN (a) and P(VDF-TrFE) (b) electrolytes after cycling.


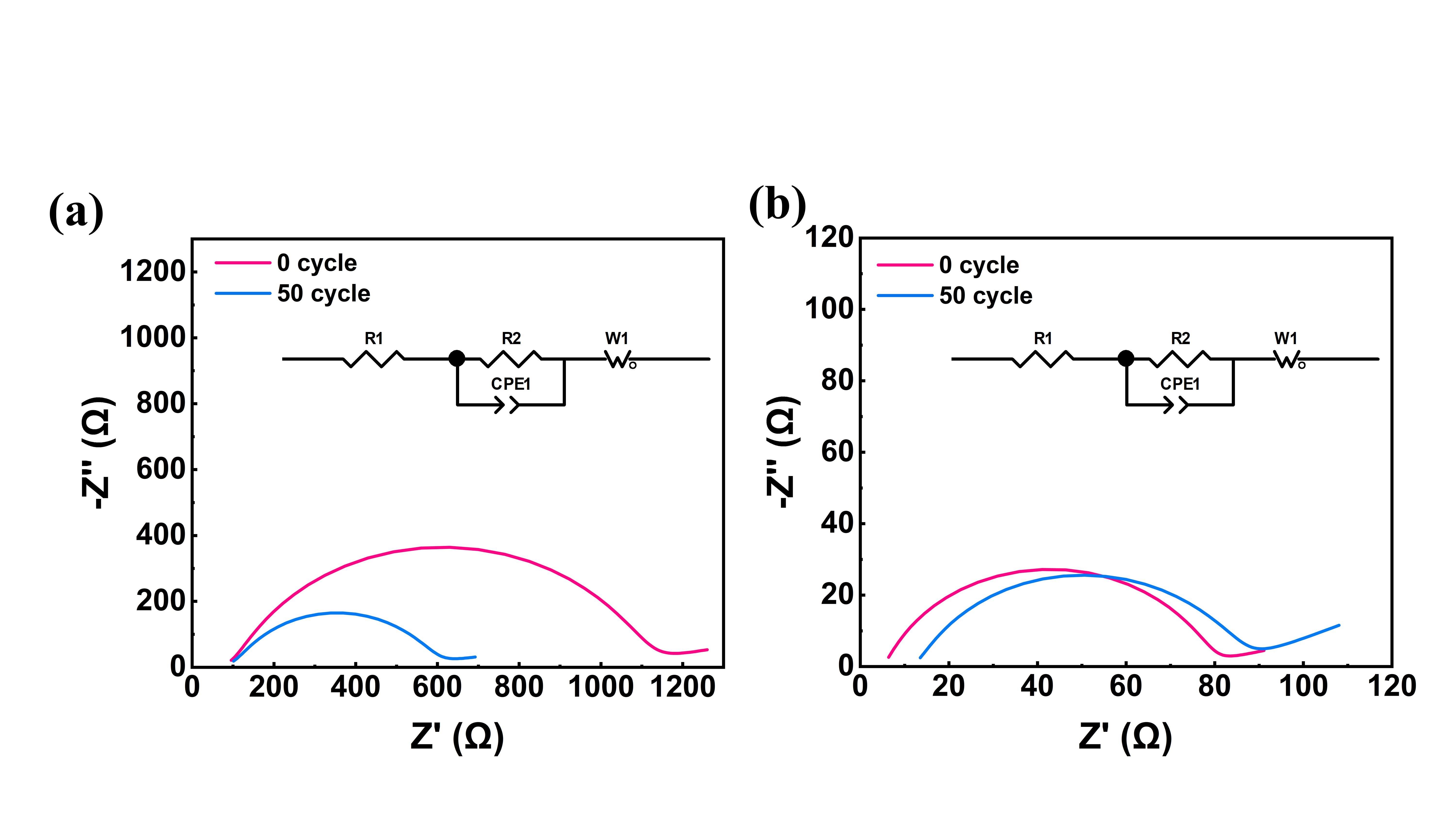


**Figure S16.** Nyquist plots of Li/P(VDF-TrFE)/Li (a) and Li/PTZN/Li cells (b) before and after cycling.


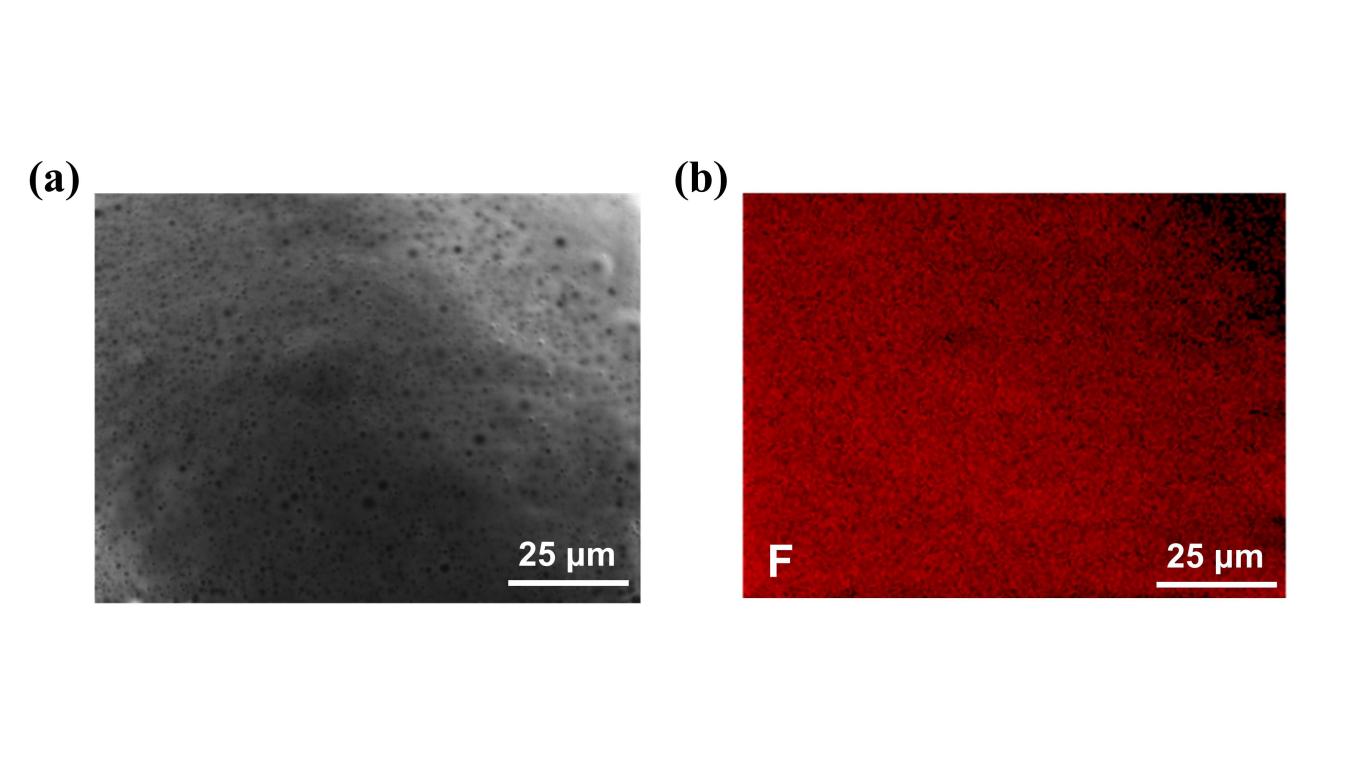


**Figure S17.** SEM (a) image and EDS (b) spectrum of the SEI layer on the lithium metal surface after cycling of Li/PTZN/Li cells.


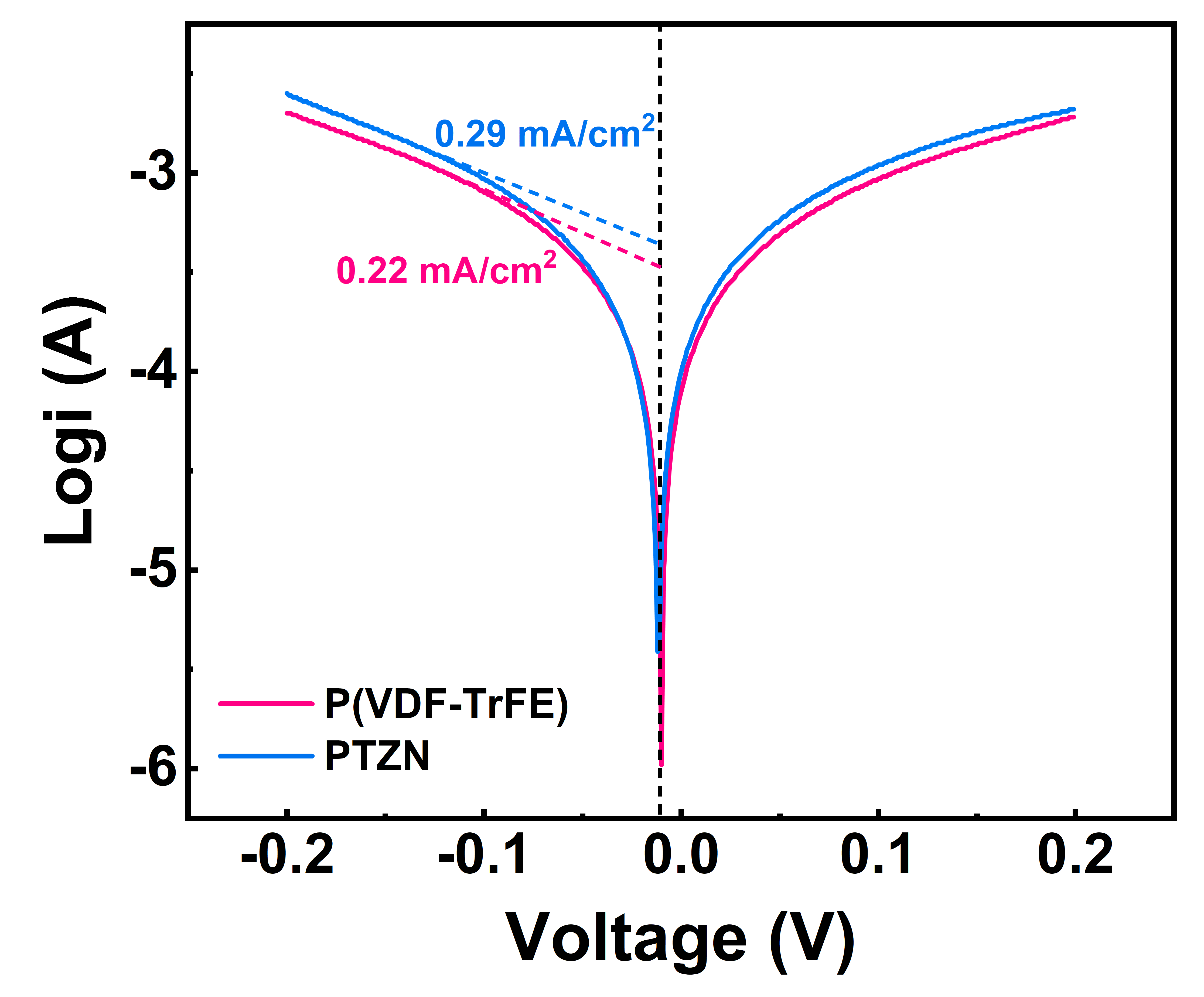


**Figure S18.** Tafel curves of Li || Li symmetric cells with P(VDF-TrFE) and PTZN electrolyte.


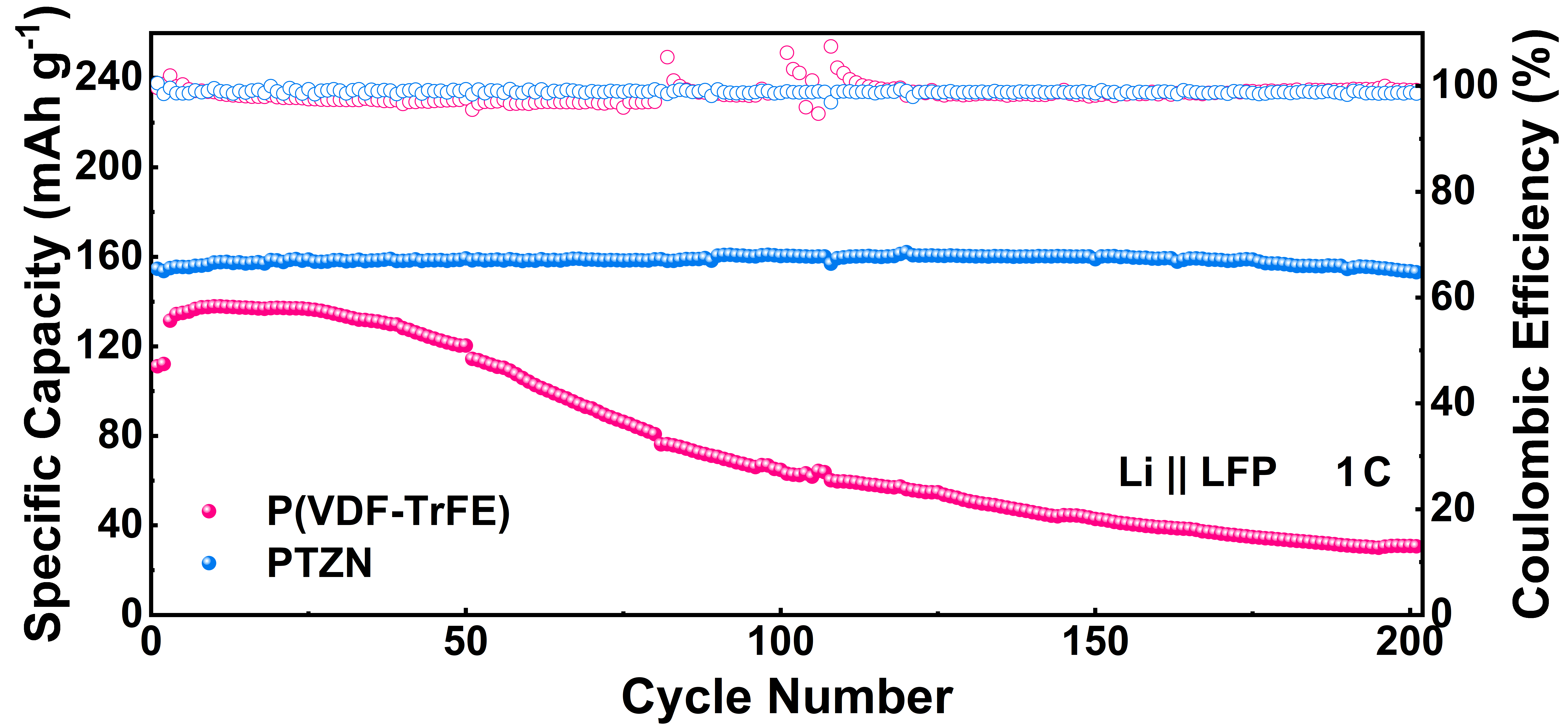


**Figure S19.** Long-cycle performance of Li || LFP cells with P(VDF-TrFE) and PTZN electrolytes at 1 C.


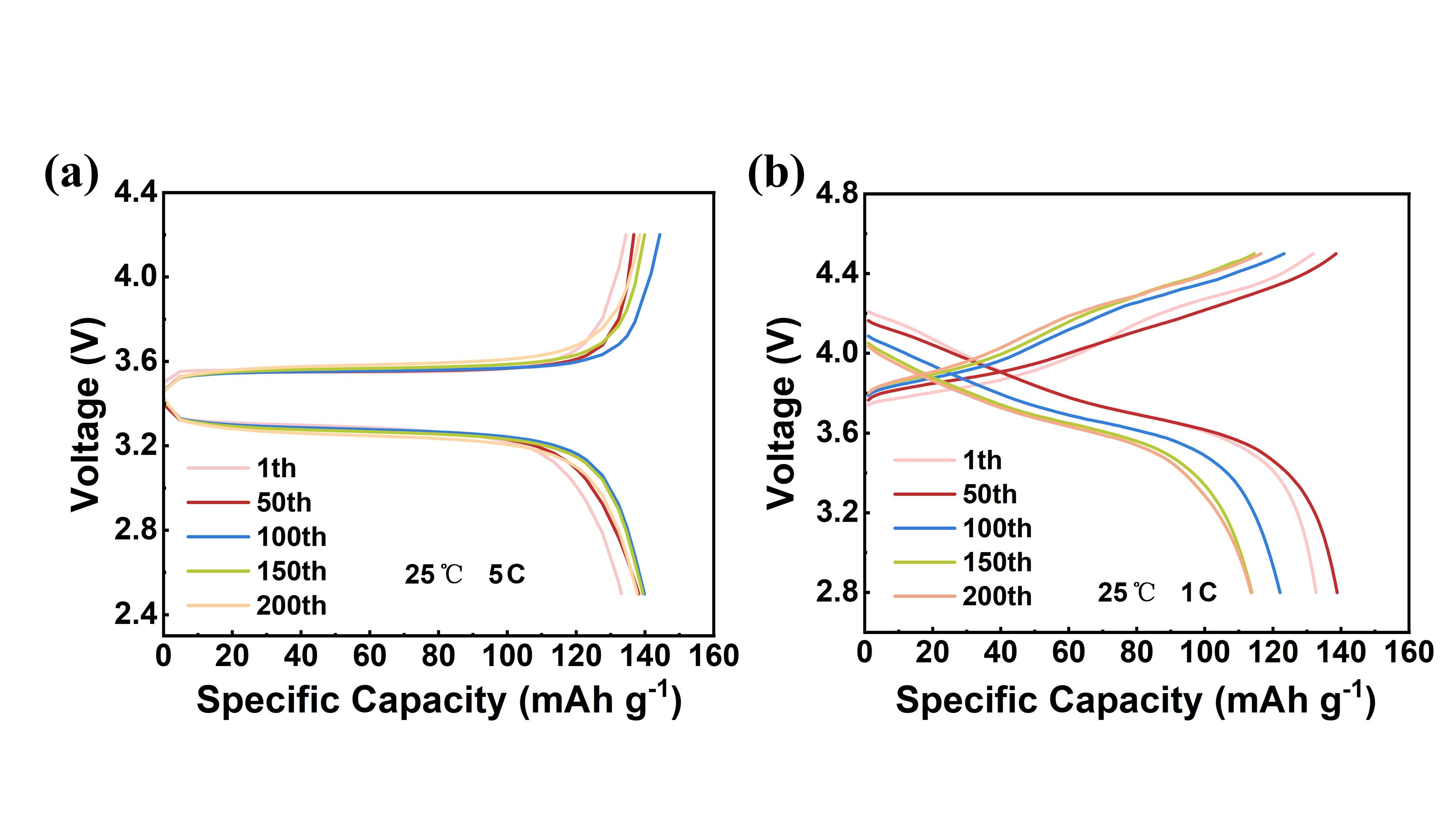


**Figure S20.** (a) Charge-discharge curve of Li/PTZN/LFP battery at 5 C. (b) Charge-discharge curve of Li/PTZN/NCM712 battery at 1 C.


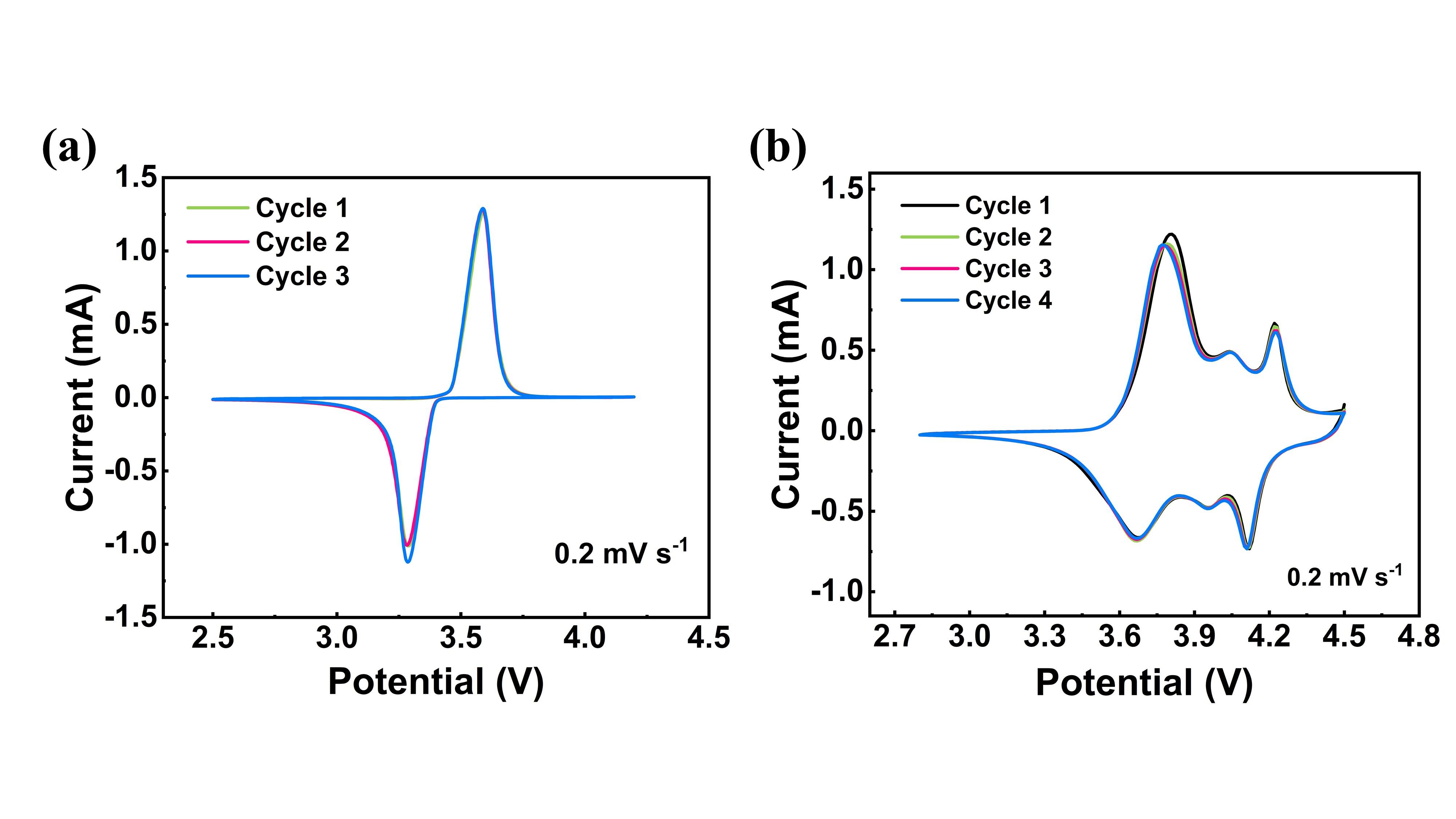


**Figure S21.** CV curves of Li/PTZN/LFP cell (a) and Li/PTZN/NCM712 cell (b) at a scan rate of 0.2 mV s^-1^.

**References**

[1] T. D. Kühne, M. Iannuzzi, M. Del Ben, V. V. Rybkin, P. Seewald, F. Stein, T. Laino, R. Z. Khaliullin, O. Schütt, F. Schiffmann, D. Golze, J. Wilhelm, S. Chulkov, M. H. Bani-Hashemian, V. Weber, U. Borštnik, M. Taillefumier, A. S. Jakobovits, A. Lazzaro, H. Pabst, T. Müller, R. Schade, M. Guidon, S. Andermatt, N. Holmberg, G. K. Schenter, A. Hehn, A. Bussy, F. Belleflamme, G. Tabacchi, A. Glöß, M. Lass, I. Bethune, C. J. Mundy, C. Plessl, M. Watkins, J. VandeVondele, M. Krack, J. Hutter, *The Journal of Chemical Physics* **2020**, *152*, 194103.

[2] J. VandeVondele, J. Hutter, *The Journal of Chemical Physics* **2007**, *127*, 114105.

[3] S. Grimme, J. Antony, S. Ehrlich, H. Krieg, *The Journal of Chemical Physics* **2010**, *132*, 154104.

[4] a)X. An, Y. Liu, K. Yang, J. Mi, J. Ma, D. Zhang, L. Chen, X. Liu, S. Guo, Y. Li, Y. Ma, M. Liu, Y.-B. He, F. Kang, *Adv. Mater.* **2024**, *36*, 2311195; b)B.-H. Kang, S.-F. Li, J. Yang, Z.-M. Li, Y.-F. Huang, *ACS Nano* **2023**, *17*, 14114; c)X. Yi, Y. Guo, S. Chi, S. Pan, C. Geng, M. Li, Z. Li, W. Lv, S. Wu, Q.-H. Yang, *Advanced Functional Materials* **2023**, *33*, 2303574; d)P. Zhai, Z. Yang, Y. Wei, X. Guo, Y. Gong, *Advanced Energy Materials* **2022**, *12*, 2200967; e)J.-H. Kim, D.-H. Park, J.-S. Jang, J.-H. Shin, M.-C. Kim, S.-B. Kim, S.-H. Moon, S.-N. Lee, K.-W. Park, *Chemical Engineering Journal* **2022**, *446*, 137035; f)H. Jiang, Y. Du, L. Zhao, X. Liu, J. Kong, P. Liu, T. Zhou, *Chemical Engineering Journal* **2024**, *487*, 150455; g)T. Wang, H. Yuan, H. Wang, Y. Guo, J. Yang, X. Liu, B. Liu, X. Wang, C. H. Kirk, J. Sun, Y.-W. Zhang, J. Wang, *Advanced Functional Materials* **2024**, *34*, 2405699; h)Z. Li, S. Wang, J. Shi, Y. Liu, S. Zheng, H. Zou, Y. Chen, W. Kuang, K. Ding, L. Chen, Y.-q. Lan, Y.-p. Cai, Q. Zheng, *Energy Storage Materials* **2022**, *47*, 262; i)X.-L. Zhang, F.-Y. Shen, X. Long, S. Zheng, Z. Ruan, Y.-P. Cai, X.-J. Hong, Q. Zheng, *Energy Storage Materials* **2022**, *52*, 201; j)T. Liu, Y. Zhong, Z. Yan, B. He, T. Liu, Z. Ling, B. Li, X. Liu, J. Zhu, L. Jiang, X. Gao, R. Zhang, J. Zhang, B. Xu, G. Zhang, *Angewandte Chemie International Edition* **2024**, *63*, e202411535.
